# Supplementary material for: Importance of sex and gender factors for COVID-19 infection and hospitalisation: a sex-stratified analysis using machine learning in UK Biobank data
Source: BMJ Open. 2022 May 18;12(5):e050450. doi: 10.1136/bmjopen-2021-050450 (PMC9118360; doi:10.1136/bmjopen-2021-050450)
Supplement: Supplementary data [file bmjopen-2021-050450supp001.pdf]

Supplemental

Supplement 1. Definition of deprivation indices

Variable Name	**Definition**
Education score	This measure shows the extent of deprivation in education, skills and training in an area within 2 sub domains including children and young people and adults skills.
Living environment score	This score measures the quality of individuals' immediate surroundings both within and outside the home within the 'indoors' living environment, which measures the quality of housing, and the 'outdoors' living environment which contains two measures relating to air quality and road traffic accidents subdomains.
Employment score	This score measures employment deprivation in an area conceptualized as involuntary exclusion of the working age population from the labour market.
Income score	This score measures the proportion of the population in an area experiencing deprivation related to low income.
Housing score	This score measures the physical and financial accessibility of housing and key local services. The indicators fall into two sub-domains: 'geographical barriers', which relate to the physical proximity of local services, and 'wider barriers' which includes issues relating to access to housing such as affordability.
Health score	This score measures premature death and the impairment of quality of life by poor health. It considers both physical and mental health. The domain measures morbidity, disability and premature mortality but not aspects of behaviour or environment that may be predictive of future health deprivation.

Supplement 2: Grid Search Parameters

**Shrinkage**	0.3	0.1	0.05	0.01	0.001
**Interaction Depth**	3	5	7		
**N.minobsinnode**	5	10	15		
**Bag.fraction**	0.65	0.5	0.8	1	

Azizi Z, *et al. BMJ Open* 2022; 12:e050450. doi: 10.1136/bmjopen-2021-050450
