# Supplementary material for: Importance of sex and gender factors for COVID-19 infection and hospitalisation: a sex-stratified analysis using machine learning in UK Biobank data
Source: BMJ Open. 2022 May 18;12(5):e050450. doi: 10.1136/bmjopen-2021-050450 (PMC9118360; doi:10.1136/bmjopen-2021-050450)
Supplement: Author's manuscript [file bmjopen-2021-050450.draft_revisions.pdf]

BMJ Open

BMJ Open is committed to open peer review. As part of this commitment we make the peer review history of every article we publish publicly available.

When an article is published we post the peer reviewers' comments and the authors' responses online. We also post the versions of the paper that were used during peer review. These are the versions that the peer review comments apply to.

The versions of the paper that follow are the versions that were submitted during the peer review process. They are not the versions of record or the final published versions. They should not be cited or distributed as the published version of this manuscript.

BMJ Open is an open access journal and the full, final, typeset and author-corrected version of record of the manuscript is available on our site with no access controls, subscription charges or pay-per-view fees (http://bmjopen.bmj.com).

If you have any questions on BMJ Open's open peer review process please email info.bmjopen@bmj.com

BMJ Open

The Importance of Sex and Gender Factors for COVID-19 Vaccination: A Sex-stratified Analysis Using Machine Learning

Journal:	*BMJ Open*
Manuscript ID	bmjopen-2021-050450
Article Type:	Original research
Date Submitted by the Author:	23-Feb-2021
Complete List of Authors:	Azizi, Zahra; McGill University Health Centre<br>Shiba, Yumika; McGill University, Department of Biology<br>Alipour, Pouria; McGill University Health Centre; McGill University<br>Maleki, Farhad; McGill University Health Centre, Department of Radiology<br>Raparelli, Valeria; University of Ferrara, Department of Translational Medicine; University of Alberta, Department of Translational Medicine<br>Norris, Colleen; University of Alberta, Faculty of Nursing<br>Forghani, Reza; McGill University Health Centre, Department of Radiology<br>Pilote, Louise; McGill University Health Centre; McGill University<br>El Emam, Khaled; University of Ottawa
Keywords:	COVID-19, Health policy < HEALTH SERVICES ADMINISTRATION & MANAGEMENT, Risk management < HEALTH SERVICES ADMINISTRATION & MANAGEMENT

SCHOLARONE™
Manuscripts

I, the Submitting Author has the right to grant and does grant on behalf of all authors of the Work (as defined in the below author licence), an exclusive licence and/or a non-exclusive licence for contributions from authors who are: i) UK Crown employees; ii) where BMJ has agreed a CC-BY licence shall apply, and/or iii) in accordance with the terms applicable for US Federal Government officers or employees acting as part of their official duties; on a worldwide, perpetual, irrevocable, royalty-free basis to BMJ Publishing Group Ltd ("BMJ") its licensees and where the relevant Journal is co-owned by BMJ to the co-owners of the Journal, to publish the Work in this journal and any other BMJ products and to exploit all rights, as set out in our licence.

The Submitting Author accepts and understands that any supply made under these terms is made by BMJ to the Submitting Author unless you are acting as an employee on behalf of your employer or a postgraduate student of an affiliated institution which is paying any applicable article publishing charge ("APC") for Open Access articles. Where the Submitting Author wishes to make the Work available on an Open Access basis (and intends to pay the relevant APC), the terms of reuse of such Open Access shall be governed by a Creative Commons licence – details of these licences and which Creative Commons licence will apply to this Work are set out in our licence referred to above.

Other than as permitted in any relevant BMJ Author's Self Archiving Policies, I confirm this Work has not been accepted for publication elsewhere, is not being considered for publication elsewhere and does not duplicate material already published. I confirm all authors consent to publication of this Work and authorise the granting of this licence.

For peer review only

The Importance of Sex and Gender Factors for COVID-19 Vaccination: A Sex-stratified Analysis Using Machine Learning

Zahra Azizi, MD, MSc [1]; Yumika Shiba [2]; Pouria Alipour, MSc[1,3]; Farhad Maleki, PhD[4]; Valeria Raparelli, MD, PhD [5,6]; Colleen Norris, PhD [6,7]; Reza Forghani, MD, PhD[4]; Louise Pilote, MD, PhD* [1,3,8] ; Khaled El-Emam, PhD* [9,10,11] and the GOING FWD Investigators

***Drs Pilote and El-Emam are co-senior authors**

1. Centre for Outcomes Research and Evaluation, McGill University Health Centre Research Institute, Montreal, QC, Canada
2. Department of Biology; McGill University, Montreal, QC, Canada
3. Faculty of Medicine and Health Sciences, McGill University, Montreal, QC, Canada
4. Augmented Intelligence & Precision Health Laboratory (AIPHL), Department of Radiology, McGill University Health Centre, Montreal, QC, Canada
5. Department of Translational Medicine, University of Ferrara, Ferrara, Italy
6. Faculty of Nursing, University of Alberta, Edmonton, Alberta, Canada
7. Heart and Stroke Strategic Clinical Networks-Alberta Health Services, Alberta, Canada
8. Divisions of Clinical Epidemiology and General Internal Medicine, McGill University Health Centre Research Institute, Montreal, QC, Canada
9. Children's Hospital of Eastern Ontario Research Institute, Ottawa, Ontario, Canada
10. Faculty of Medicine, University of Ottawa, Ottawa, Ontario. Canada
11. Replica Analytics Ltd, Ottawa, Ontario, Canada

Corresponding Authors:

Louise Pilote MD, MPH, PhD
Center for Outcomes Research and Evaluation
Divisions of Clinical Epidemiology and General Internal Medicine
McGill University Health Centre Research Institute
5252 boulevard de Maisonneuve, Montreal
Quebec, H3A 1A1, Canada
Tel: 514 934-1934 ext. 44722; Fax: 514 843-1676
E-mail: louise.pilote@mcgill.ca

Khaled El Emam, PhD
Children's Hospital of Eastern Ontario Research Institute
401 Smyth Road, Ottawa
Ontario K1J 8L1, Canada
E-mail: kelemam@ehealthinformation.ca
Tel: +1 613-797-5412

Word Count: 3169 (w/o abstract and references)

For peer review only - http://bmjopen.bmj.com/site/about/guidelines.xhtml

Abstract:

Objective: To examine sex and gender roles in COVID-19 test positivity and hospitalization in sex-stratified predictive models using machine learning.

Design: Cross-sectional study

Setting: UK Biobank prospective cohort

Participants: Participants tested between March 16th, 2020 to May 18th, 2020 were analyzed.

Main outcome measures: The endpoints of the study were COVID-19 test positivity and hospitalization. Forty-two individuals' demographics, psychosocial factors, and comorbidities were used as likely determinants of outcomes. Gradient boosting machine was utilized for building prediction models.

Results: Of 4,510 individuals tested (51.2% females, mean age= 68.5±8.9 years), 29.4% were test positive. Males were more likely to be positive than females (31.6% vs 27.3%, P=0.001). In females, living in more deprived areas, lower income, increased LDL-to-HDL ratio, working night shifts, and living with a greater number of family members were associated with a higher likelihood of COVID-19 positive test. While in males, greater body mass index and LDL-to-HDL ratio were the factors associated with a positive test. Older age, and adverse cardio-metabolic characteristics were the most prominent variables associated with hospitalization of test positive patients in both overall, and sex-stratified models.

Conclusion: High-risk jobs, crowded living arrangements, and living in deprived areas were associated with increased COVID-19 infection in females, while high-risk cardio-metabolic characteristics were more influential in males. Gender-related factors should be considered in identifying priority groups for COVID-19 infection vaccination campaigns, especially in females.

Keywords: Covid-19 infection, SARS-Cov-2, Test Positive, Hospitalization, Sex, Gender, Machine

For peer review only - http://bmjopen.bmj.com/site/about/guidelines.xhtml

Learning, Vaccination

Strengths and limitations:

- A unique feature of the study is the investigation of numerous psycho-socio-cultural factors in conjunction with clinical and laboratory factors made feasible through machine learning algorithms.
- The assessment of sex-stratified algorithms to elucidate the most influential factors in both sexes adds novel information as it has rarely been done to date.
- The first limitation of this study is selection bias due to the lack of systematic and random testing across the UK.
- This analysis was done using the baseline diagnostic data that was collected from 2006 to 2010; therefore, misclassification of determinants is likely. However, previous studies of the UK Biobank have shown a high correlation between baseline and follow-up data for a subsample of patients who had further visits for imaging.
- Finally, our relatively lower predictive model performance—as expected—reflects other influences not captured in the UK Biobank. A recent study in UK Biobank revealed similar performance in the gbm model which further illustrates the accuracy of our findings.

For peer review only

Introduction

The novel coronavirus SARS-CoV-2 (COVID-19) pandemic has led to more than 105 million reported positive cases and over two million deaths worldwide [1]. As vaccination is rolling out, continuous efforts are made to establish risk factors for the disease and find vulnerable populations across the globe [2-8].

There has been an imbalance in infection susceptibility, severity, and mortality between males and females [9]. These differences are multifactorial and have been attributed to a combination of biological (i.e. genetic, hormonal) and psycho-socio-cultural differences (gender) [9-13]. While "sex" refers to a set of biological attributes in humans and animals, "gender" refers to the roles, behaviours, and identities of individuals that forms throughout life [14 15]. It is increasingly recognized that both sex and gender play significant roles in health outcomes [14 15], including the acquisition of infections and response to infection and treatments [16]. The world health organization statement [16], addressing sex and gender in epidemic-prone infectious diseases, outlines that differences between males and females can lead to differences in activity patterns in work and in family roles, which may increase the risk of exposure to infectious disease in a particular setting [16]. Therefore, lack of consideration of the influences of sex and gender in COVID-19 contraction can potentially hinder the effectiveness of COVID-19 vaccination prioritization strategies.

With the introduction of the COVID-19 vaccines, identifying those most vulnerable to infection with accurate prediction models that account for more complex relationships is urgent. Machine learning algorithms can explore non-linear and complex relationships by considering the interaction between both biological and psycho-socio-cultural factors together; however, these methods are still underutilized in the medical field, and few predictive models have been tailored to each of the sexes [17-19]. Therefore, we examined sex and gender-related factors associated with

For peer review only - http://bmjopen.bmj.com/site/about/guidelines.xhtml

SARS-CoV-2 test positivity and COVID-19 hospitalization in the UK Biobank cohort and developed sex-stratified predictive models using machine learning methods.

Methods

Study Population

This is a cross sectional study of UK Biobank study. The UK Biobank (UKB, https://www.ukbiobank.ac.uk/) is a prospective cohort study that collects health, lifestyle, genetic, and imaging data for over 500,000 randomly selected participants in the UK [20]. Baseline data collection took place between 2006–2010 across England, Scotland, and Wales. The age of participants at recruitment was between 40 and 69 years [20]. Data was collected in 22 Assessment Centers through 4 main methods; touchscreen questionnaires, verbal interviews, physical measures, and biological sampling [20]. For this study, only data from England between March 16th, 2020 to May 18th, 2020 was used due to the unavailability of COVID-19 test results from Scotland and Wales at the time of analysis.

Ethics approval for the project was obtained from the coordinator center at McGill University, Canada (CIHR GNP-161904, 2018/10/01 to 2023/03/31). The data was obtained from UKBiobank repository (Application ID:45551).

Patient and Public Involvement

No patient involved in the design, or conduct, or reporting, or dissemination plans of our research.

COVID-19 Test Results

COVID-19 test results were available from Public Health England (PHE) data, which was linked to UK Biobank baseline data [21]. The primary endpoint of the study was test positivity. We

For peer review only - http://bmjopen.bmj.com/site/about/guidelines.xhtml

defined testing positive as having at least one positive test result. The test results were available from March 16th, 2020 to May 18th, 2020. The secondary endpoint of the study was being hospitalized for a COVID-19 related illness. For this purpose, we chose test-positive patients who had at least one positive result in an in-patient setting. The results are from samples taken from the combined nose, throat, sputum, or lower respiratory tract. The analysis for infection was done using real-time polymerase chain reaction (RT-PCR) tests for SARS-CoV-2.

Baseline Characteristics

Patients' demographics, psychosocial (gender-related variables), anthropometric variables, and comorbidities that were collected at baseline 2006-2010 were used for analysis. We used this particular baseline data for socioeconomic status and occupation since studies have shown a high correlation between baseline and follow-up data for a subsample of patients who had further visits for imaging [22].

Gender-related Psychosocial Variables

A multistep approach for identifying gender-related variables was exploited based on GOING-FWD (Gender Outcomes International Group to Further Well-being Development) methodology [23], the Women Health Research Network's gender framework (i.e. gender identity, gender roles, gender relationships, and institutionalized gender), and the Canadian Institutes of Health Research sex and gender modules [14 15 24]. Based on this approach and data availability, the following variables were selected: multiple deprivation indices (Supplemental 1), employment, jobs involving night shift, education level, smoking status, alcohol consumption, number of children, household crowding, housing ownership, income, leisure and social activities, risk-taking

For peer review only - http://bmjopen.bmj.com/site/about/guidelines.xhtml

behaviors, and neuroticism score. Details of the variables are available in the UK Biobank data dictionary (https://biobank.ctsu.ox.ac.uk/crystal/search.cgi).

Comorbidities

Baseline comorbidities were self-reported and collected using a touch screen device—i.e. hypertension (HTN), diabetes (DM), chronic obstructive pulmonary disease (COPD), asthma, allergy, history of stroke, heart attack, angina, deep vein thrombosis (DVT), pulmonary embolism (PE), and cancer (CA). In addition, the number of medications and long-standing illness, disability, or infirmity were also included and coded as dichotomous variables.

Physical and Biological Characteristics

Variables used as measures of physical and biological characteristics were body mass index (BMI) (defined as weight (Kg)/height $(m)^2$), waist to hip ratio (WHR), levels of vitamin D, hemoglobin A1C (HbA1C), high-density lipoprotein (HDL) and low-density lipoprotein (LDL); all coded as continuous variables.

Statistical Analyses

Descriptive statistics were reported as mean and standard deviation for continuous variables and frequency and percentage for categorical variables. Group-based differences (test negative versus positive) in baseline characteristics were compared using independent Student's t-test for continuous variables (or its non-parametric counterpart in skewed distribution) and chi-square for categorical variables. P-values of less-than or equal-to 0.05 were considered statistically significant. Case analysis (pairwise deletion) approach was used for dealing with missing data. Data analysis was performed using R-Studio (Version 1.3.1093) and R software (v4.0.3).

Machine Learning

For peer review only - http://bmjopen.bmj.com/site/about/guidelines.xhtml

The data was split into 70% training and 30% test sets, where the test set was only used for the evaluation of the final models. The training set was used to develop gradient boosting machine models [25-27] using "gbm3" R package for predicting SARS-Cov-2 test-positivity and hospitalization prediction. To alleviate the effect of class imbalance on the model development [28], an oversampling approach was used. For each endpoint, i.e. test-positivity or hospitalization, three different models were developed: (1) a model for males, (2) a model for females, and (3) an overall model for males and females combined.

Using k-fold cross-validation is considered a best practice in developing machine learning models [29]. Therefore, for developing each model, a 10-fold cross-validation was performed on the training set. We also used a grid-search procedure to find the best combination of boosting hyper-parameters and tree specific hyper-parameters (e.g. learning rate (shrinkage), tree depth, bag fraction, and the minimum number of observations in terminal nodes). Bernoulli distribution was used for classification models. These resulted in three trained models for SARS-Cov-2 test-positivity and three models for SARS-Cov-2 hospitalization.

To provide an unbiased estimate of the model generalization errors, the performance of the trained models was assessed and reported on the test set. Confusion matrix derived metrics including accuracy, precision, recall (sensitivity), specificity as well as AUC score were used as the performance measures [30].

Most Influential Variables

For classifying the most influential variables, the permutation-based feature importance was utilized [31]. This approach measures a feature (variable) importance by calculating the increase of the model's prediction error after permuting the feature.

We reported partial dependence plots (PDPs) using "pdp" package in R to understand the

For peer review only - http://bmjopen.bmj.com/site/about/guidelines.xhtml

marginal effect of a feature on the predicted outcome. PDP demonstrates how the response variable changes as we change the value of a feature while taking into account the average effect of all the other features in the model [32].

Results

Of 4,510 patients (51.2% females, and 68.5±8.88 years) who were tested, 29.4% were positive. Females were less likely to be positive (males:31.6% versus females:27.3%, P=0.001). In descriptive analyses, amongst all patients, those older than 80 years, minority ethnicities, and individuals born outside of the UK had a higher infection rate. Moreover, infected patients had higher BMI, WHR, HBA1C, LDL to HDL ratio, and lower HDL levels. On the other hand, smokers, alcohol drinkers, more educated individuals, house owners, and those who did not participate in any social activity had a lower rate of infection. Test positive patients tended to have a greater number of people in their household and live in more deprived areas. This relationship held true in a sex-stratified analysis (Tables 1 and 2).

ML-based Prediction Models for SARS-Cov-2 Positive Test

The performance of the gradient boosting machine models is summarized in Table 3. The prediction models for the overall study population suggest that an increased LDL to HDL ratio, WHR, and age were associated with a higher likelihood of test positivity. Additionally, individuals who worked night shifts or lived in a more deprived area (lower environment and education scores) as well as those participating in social activities—including sports clubs, bars, and religious groups—had a higher risk of having a positive test. In contrast, individuals with higher education levels, higher income, and those with daily or almost daily alcohol consumption were less likely to have a positive result. Figure 1 illustrates the partial dependence plots used for interpreting the results and direction of each variable in the models.

For peer review only - http://bmjopen.bmj.com/site/about/guidelines.xhtml

Sex-specific models for test positivity showed that gender factors were more important in females, whereas in males, biological factors were significant contributors to test positivity. Females who lived in more deprived areas (increased environment score), had increased LDL to HDL ratio, worked night shifts, and had a greater number of family members in their household were more likely to test positive. Moreover, those with income greater than 100K were less likely to test positive. (Figure 2). In comparison, males with greater BMI and LDL to HDL ratio, more deprived area (greater heath score) and black ethnicity were more likely to test positive (Figure 3).

ML-based Prediction Models for COVID-19 Related Hospitalizations

The performance of the gradient boosting machine models is summarized in Table 4. Amongst the 1,326 test-positive patients, 932 (70.3%) were hospitalized (females: 413 (44.3%)). The result of the overall model to predict hospitalization in test-positive patients revealed that those with higher HbA1c level, older age, greater BMI, higher LDL to HDL ratio and greater number of medications had greater risk of being hospitalized (Figure 4).

Sex stratified model revealed older age, a higher level of HbA1c, LDL to HDL ratio, greater number of medications and higher-housing score (showing more deprived areas) were most influential variables in predicting hospitalization in test-positive females (Figure 5), whereas older age, an increased HbA1c level, WHR, LDL to HDL ratio and BMI were the most influential variables associated with hospitalization in test-positive males (Figure 6).

Discussion

The present evaluation of individuals tested for SARS-CoV-2 in a UK cohort demonstrates the importance of gender-related factors along with clinical characteristics in predicting COVID-19 test positivity, hence providing guidance for identifying vaccination priority groups in the general population. Whilst factors related to the gender role of individuals

For peer review only - http://bmjopen.bmj.com/site/about/guidelines.xhtml

were the most influential determinants in females, cardio-metabolic risk factors played a key role in males. Such a sex-specific cluster of factors associated with adverse outcomes was attenuated when considering the rate of COVID-19 related hospitalization among test-positive individuals. Notably, older age and cardio-metabolic diseases, including diabetes, obesity, and dyslipidemia, were most influential regardless of sex.

Emerging evidence has shown sex differences in contracting and severity of the infection. While most investigations have focused on biological factors as the potential culprit, few have incorporated gender determinants. Various modulating mechanisms have been suggested, including genetic factors (hormone-regulated expression of genes), the difference in innate and adaptive immune responses, as well as gendered factors such as lifestyle, behavioral and psychosocial factors [11 12 33].

Our findings reinforce and advance the current evidence related to the impact of metabolic comorbidities and older age in the COVID-19 pandemic. Obesity has been linked with impaired pulmonary function and suppression of immune response and has been recognized as one of the most important factors in contracting COVID-19 infection, the severity of the disease, and mortality [4 34]. Higher BMI and WHR are some of the more influential measures that have shown a dose-response relationship with test positivity and disease severity [4 6 34]. A study in UK biobank demonstrated a more than 50 percent increase in COVID-19 infection in obese and severely obese patients compared to non-obese individuals [34]. Investigation of different databases in various countries have also reported older age and comorbidities as the most important factors associated with clinical severity and mortality [35]. Specifically, diabetes has been associated with more severe disease manifestation [36]. This is consistent with our results, that demonstrated HbA1c level as one of the most prominent factors for hospitalization in the overall, and sex-

For peer review only - http://bmjopen.bmj.com/site/about/guidelines.xhtml

stratified models. Preliminary studies have demonstrated the association between HDL level, infection, and disease severity. While the mechanism of this correlation is unknown, this can be due to the antioxidant, antithrombotic and anti-inflammatory role of HDL cholesterol [37].

A major and novel contribution of this study is the application of a sex and gender-oriented lens to inform the understanding of COVID-19 infection by conducting sex-disaggregated analyses and incorporating both sex and gender-related determinants. Studies have shown socioeconomic disadvantages such as living in a more deprived area and lower education to be associated with increased risk of infection and disease severity [2 4]; these factors prevail in individuals more likely to work in service-based occupations, be self-employed, or live in crowded households [3 4 38]. Moreover, less access to healthcare is another factor that leads to greater infection risk and worst outcomes. By the same token, our study demonstrated that those who live in a more deprived area with lower SES and education were more susceptible to COVID-19 infection, but the impact of gender determinants was more significant amongst females. Women's role as caregivers within family and society increases their risk of infection [33]. Moreover, women are more likely to work as frontline workers, including nursing positions, increasing their exposure to the virus [3 22 33]. In our current study, environment score, household arrangement, working night shifts, and income were amongst the most important factors for females. In contrast, obesity, LDL to HDL ratio, and alcohol consumption were amongst the most influential factors in men.

The results of this study serve as an important guide for vaccination prioritization policies. While essential workers and elderly individuals have already been targeted for vaccination, the next step will be the identification and prompt vaccination of individuals in higher-risk groups in the general population. Although factors such as diabetes and obesity

For peer review only - http://bmjopen.bmj.com/site/about/guidelines.xhtml

might be important, psychosocial risk factors such as lower socioeconomic status, education level, living in a more deprived environment, risky occupation, and household crowding should also be taken into account. Individuals, especially females, exhibiting such high-risk gendered factors should be prioritized for vaccination.

Strengths and Limitations

A unique feature of the study is the investigation of numerous lifestyles, socioeconomic, mental, and behavioral factors representing different dimensions of gender in conjunction with clinical and laboratory factors made feasible through machine learning algorithms. Furthermore, the assessment of sex-stratified algorithms to elucidate the most influential factors in both sexes adds novel information as it has rarely been done to date.

The study should also be interpreted in light of some limitations. The first limitation of this study is selection bias due to the lack of systematic and random testing across the UK. This analysis was done using the baseline diagnostic data that was collected from 2006 to 2010; therefore, misclassification of determinants is likely. However, previous studies of the UK Biobank have shown a high correlation between baseline and follow-up data for a subsample of patients who had further visits for imaging [22]. Moreover, a disproportionately higher representation of Caucasians in the study may make the results less generalizable to other ethnic groups. Finally, though we attempted to test various clinical, social and demographic factors to predict test positivity, it is essential to note that acquisition of infection is a multifactorial phenomenon that cannot be easily encoded using a small set of variables. Our relatively low predictive model performance—as expected—reflects other influences not captured in the UK Biobank. Similar results were obtained using the XGboost method from a recent study on the UK Biobank dataset whereby similar performance to our gbm model was obtained, which further

For peer review only - http://bmjopen.bmj.com/site/about/guidelines.xhtml

supports the interpretation that this is the expected accuracy for predictive models on this dataset[18].

Conclusions

Sex-specific risk patterns of COVID-19 test-positivity exist, with gender-related factors being more relevant in females and biological factors in males. Specifically, socioeconomic status, education level, number of people living in a household, and high-risk jobs were associated with a higher likelihood of contracting the infection in female individuals, whereas cardiometabolic disease and obesity were more associated in males. The rate of COVID-19 related hospitalization recognized similar favoring clinical factors regardless of sex. This study highlights the importance of prioritizing high-risk groups using psychosocial determinants along with clinical factors as a targeted approach for vaccination of more at-risk population to contain SARS-Cov-2 pandemic.

Funding: The GOING-FWD Consortium is funded by the GENDER-NET Plus ERA-NET Initiative (Project Ref. Number: GNP-78): The Canadian Institutes of Health Research (GNP-161904), "La Caixa" Foundation (ID 100010434) with code LCF/PR/DE18/52010001, The Swedish Research Council (2018-00932) and The Austrian Science Fund (FWF, I 4209). VR was funded by the Scientific Independence of Young Researcher Program of the Italian Ministry of University, Education, and Research (RBSI14HNVT).

Contributors:

Drs Pilote and El-Emam contributed equally as co-senior authors. ZA, YS designed the study, developed the questions and contributed to the development of conceptual framework and writing the first draft. ZA performed the main analysis and critical revisions of manuscript. FM and RF validated the results by performing secondary analysis and finalizing the draft. LP, CN, VR, KEE supervised the project from study design to final draft and approved the final version

For peer review only - http://bmjopen.bmj.com/site/about/guidelines.xhtml

of manuscript.

Disclosures:

The authors disclose no conflict of interest related to this study.

For peer review only

For peer review only - http://bmjopen.bmj.com/site/about/guidelines.xhtml

Table 1. Descriptive characteristics of COVID-19 test positive versus negative males and females

Baseline Characteristics	Overall				Females			Males		
	All N=4510	Test - N=3148	Test + N=1326	P	Test - N=1679	Test + N=630	P	Test - N=1505	Test + N=696	P
**Demographics**										
**Age, years**										
	68.5±8.9	68.7±8.7	67.9±9.2	0.007	67.51±8.9	66.2±9.4	0.002	70.0±8.3	69.4±8.8	0.1
**Age Group, %**										
<60	23.6	21.8	28	<0.001	26.4	34.1	0.001	16.5	22.4	0.006
>=60 - <70	24.7	25.4	23		26.6	25.6		24	20.7	
>=70 -<80	45.9	47.1	42.8		42	35.1		52.9	49.9	
>=80	5.9	5.7	6.2		4.9	5.2		6.6	7	
**Sex, %**										
Female	51.2	52.7	47.5	0.001						
**Ethnicity, %**										
White	90.7	92.4	86.8	<0.001	91.5	86.4	<0.001	93.4	87.1	<0.001
Mixed	2.3	2.2	2.4		2.6	2.2		1.7	2.6	
Asian	3.3	2.6	5		2.1	5.3		3.1	4.8	
Black	3.7	2.9	5.8		3.8	6.1		1.9	5.5	
**Country of Birth, %**										
United Kingdom	88.9	90.2	85.7	<0.001	89.1	84	0.001	91.5	87.4	0.003
**Biological Variables**										
**Number of Medications**										
	3.3±3.3	3.3±3.3	3.2±3.3	0.7	3.2±3.2	3.2±3.3	0.8	3.3±3.4	3.3±3.3	0.6
**BMI (Kg/m2)**										
	28.4±5.4	28.2±5.4	28.9±5.4	<0.001	28.1±5.8	28.7±6.2	0.02	28.3±4.9	29.1±4.7	<0.001
**WHR**										
	0.9±0.1	0.88±0.09	0.89±0.09	<0.001	0.83±0.07	0.84±0.07	0.03	0.95	0.95	0.1
**HTN, %**										
	35.3	34.5	37	0.1	28.8	31.7	0.1	41	41.7	0.7
**Stroke, %**										
	3	3	3	1	1.7	2.1	0.6	4.4	3.8	0.5
**Heart Attack, %**										
	4	4.1	3.8	0.7	1.4	1.8	0.6	7.1	5.7	0.2
**Angina, %**										
	5.7	5.9	5.3	0.4	3.3	3.4	1	8.8	7.1	0.2
**DVT, %**										
	3.3	3.1	4	0.1	2.9	4.1	0.1	3.3	3.8	0.6
**PE, %**										
	1.7	1.9	1.1	0.09	1.9	1.4	0.6	1.9	0.9	0.09
**COPD, %**										
	3.2	3.2	3.1	0.9	2.6	2.6	1	3.9	3.6	0.8
**Asthma, %**										
	13.5	13.6	13	0.5	16.4	16.3	0.9	10.5	10	0.7
**Allergy, %**										
	21.9	22.8	19.8	0.03	26.2	22.6	0.08	19	17.3	0.3
**Diabetes, %**										
	9.8	9.6	10.3	0.5	7.4	8	0.7	12	12.4	0.8

For peer review only - http://bmjopen.bmj.com/site/about/guidelines.xhtml

**Cancer, %**										
	9.4	10.1	7.7	0.01	11.1	9.2	0.2	9.1	6.4	0.4
**Disability, %**										
	45.6	46.1	44.3	0.2	42.2	38.9	0.1	50.3	49.2	0.6
**Vitamin D level**										
	46.5±21.2	46.6±21	46.1±21.6	0.4	45.7±20.7	45.2±20.9	0.6	47.6±21.4	46.7±22.2	0.4
**HbA1C level**										
	37.3±8.5	37.1±8.3	37.8±9.1	0.03	36.6±7.7	36.7±8.1	0.7	37.7±8.9	38.7±9.8	0.02
**LDL level**										
	3.4±0.88	3.4±0.8	3.4±0.8	0.7	3.4±0.8	3.5±0.8	0.5	3.3±0.8	3.3±0.8	0.8
**HDL level**										
	1.4±0.3	1.4±0.3	1.3±0.3	<0.001	1.5±0.3	1.4±0.3	<0.001	1.3±0.3	1.2±0.2	0.004
**LDL to HDL Ratio**										
	2.6±0.9	2.5±0.9	2.7±0.8	<0.001	2.3±0.8	2.5±0.8	<0.001	2.8±0.9	2.8±0.8	0.3

BMI: Body Mass Index, WHR: Waist to Hip Ratio, HTN: Hypertension, DVT: Deep Vein Thrombosis, PE: Pulmonary Embolism, COPD: Chronic Obstructive Pulmonary Disease, HbA1c: Hemoglobin A1c, LDL: Low Density Lipoprotein, HDL: High Density Lipoprotein

For peer review only

For peer review only - http://bmjopen.bmj.com/site/about/guidelines.xhtml

Table 2. Gender variables in patients who tested positive compared to test-negative patients in overall, males and females

Gender Variables,	Overall				Female			Male		
	All<br>N=4474	Test -<br>N=3161	Test +<br>N=1313	P	Test -<br>N=1668	Test +<br>N=625	P	Test -<br>N=1493	Test +<br>N=688	P
**Behavioral/Lifestyle Risk Factors**										
**Smoking, %**										
0=Never	48.5	48.3	49	0.02	54.5	58.7	0.1	41.3	40.1	<0.001
1=Previous	38.4	37.8	40		34.5	30.9		41.5	48.3	
2=Current	13.1	14	11		11	10.4		17.2	11.6	
**Alcohol, %**										
0=never	11.6	10.9	13.3	<0.001	11.8	16.4	0.001	9.9	10.6	0.002
1=occasionally	14.1	14.1	14.2		18.4	18.3		9.3	10.4	
2=1 o 3 times/month	11.8	11	13.9		14	16.9		7.7	11.1	
3=1-2/week	24.1	23.6	25.4		24.1	23.8		23.1	26.8	
4=3-4/week	19	19.6	17.4		16.2	14.1		23.4	20.4	
5=daily/almost daily	19.4	20.8	15.8		15.6	10.5		26.7	20.7	
**Gender Related Variables**										
**Employment Status, %**										
Employed	50.4	49.6	52.4	0.09	53.7	58.6	0.04	45	46.7	0.5
**Night Shift, %**										
0: Never/rarely	45.1	47.5	41.4	0.21	49	40.9	0.17	45.6	42	0.8
1: Sometimes	32.1	31.6	33		31.5	32.5		31.7	33.6	
2: Usually/Always	22.8	20.9	25.6		19.5	26.6		22.8	24.4	
**Number of Children**										
	1.9±1.4	1.8±1.4	1.9±1.4	0.03	1.8±1.2	1.9±1.2	0.15	1.8±1.5	1.9±1.6	0.12
**Education, %**										
None/	36.4	34.4	41.3	<0.001	31.2	37.5	0.02	38	44.8	0.02
NVQ/HND/HNC/CSEs	19.2	19.8	17.7		20.8	19.2		18.6	16.3	
O-Level/ GCSEs	9.9	9.9	9.8		10.5	10.6		9.2	9.1	
A-Levels/AS-Levels	34.5	35.9	31.2		37.5	32.6		34.2	29.8	
College/University/ Professional										
**Deprivation indices:**										
Education Score										
	18.6±17.9	17.7±17.4	20.6±19.3	<0.001	17.6±17.1	20.9±19.3	<0.001	17.9±17.6	20.4±18.9	0.003
Environmental Score										
	20.6±16.4	20±15.7	22.1±17.8	<0.001	19.9±15.6	21.3±17.9	0.08	20.1±15.9	22.7±17.6	0.001
Employment Score										
	0.1±0.06	0.09±0.06	0.1±0.07	<0.001	0.09±0.06	0.1±0.07	<0.001	0.101±0.06	0.109±0.07	0.01
Health Score										
	0.09±0.9	0.05±0.9	0.18±0.8	<0.001	0.05±0.8	0.15±0.9	0.02	0.06±0.9	0.22±0.08	<0.001
Housing Score										
	19.7±10.2	19.8±10.3	19.7±9.9	0.7	19.8±10.2	19.9±9.9	0.7	19.8±10.5	19.4±9.9	0.4
Income Score										
	0.13±0.11	0.13±0.1	0.15±0.11	<0.001	0.13±0.1	0.15±0.12	<0.001	0.13±0.11	0.15±0.11	0.008
**Income, %**										

For peer review only - http://bmjopen.bmj.com/site/about/guidelines.xhtml

1= <18K	32	31.5	33.5	0.1	31.2	32.1	0.17	31.8	34.7	0.26
2=18-30.9K	24.6	24.5	24.6		23.3	24		25.8	25.2	
3=31-51.9K	22.3	22.2	22.6		23.2	26.5		21.1	19.1	
4=52-100K	16.5	16.7	16		17.5	14.3		16	17.5	
5=>100K	4.6	5.1	3.3		4.8	3.2		5.3	3.5	
**Number in Household**										
	2.4±1.5	2.3±1.5	2.5±1.3	0.001	2.4±1.8	2.5±1.4	0.04	2.3±1.1	2.4±1.3	0.006
**House Ownership, %**										
	81.4	82.2	79.6	0.04	84.5	80.4	0.02	79.6	78.8	0.7
**Neuroticism Score**										
	4.3±3.3	4.3±3.3	4.2±3.3	0.4	4.8±3.3	4.8±3.2	0.9	3.8±3.2	1.7±3.3	0.5
**Risk Taking Behavior, %**										
	30.7	29.9	32.5	0.1	23.3	27	0.7	37.3	37.5	0.9
**Leisure/Social Activities, %**										
0=No	32.4	32.7	31.8		32.9	34.3	0.03	32.4	29.6	0.09
1=Sports Club or Gym	24.1	24.6	23		25.8	21.9		23.2	24.1	
2=Pub or Social Club	21.1	20.4	22.6		13.6	16.1		28	28.6	
3=Religious Group	10.4	9.5	12.5		11.5	14.8		7.3	10.4	
4=Adult Education Class	2.8	3.1	2.2		4.4	3.7		1.5	0.9	
5= Other Group Activity	9.1	9.7	7.8		11.8	9.3		7.5	6.4	

For peer review only

For peer review only - http://bmjopen.bmj.com/site/about/guidelines.xhtml

Table 3. Summary of GBM model performance for predicting test-positive results in training (10-fold cross-validation) and test sets

		Accuracy	Precision	Recall	AUC	Specificity
**Overall**	Test Set	61	0.35	0.45	0.597(0.563-0.630)	0.66
	Cross-Validation	70.22	0.38	0.51	0.603	0.64
**Male**	Test Set	60	0.37	0.43	0.589(0.532-0.645)	0.67
	Cross-Validation	68.37	0.37	0.51	0.563	0.60
**Female**	Test Set	62	0.30	0.34	0.562(0.504-0.620)	0.71
	Cross-Validation	72.51	0.40	0.39	0.597	0.77

Table4. Summary of GBM model performance for predicting being hospitalized in test-positive patients in training (10-fold cross-validation) and test sets

		Accuracy	Precision	Recall	AUC	Specificity
**Overall**	Test Set	65	0.77	0.71	0.628(0.569-0.6884)	0.5
	Cross-Validation	69.64	0.77	0.66	0.598	0.52
**Male**	Test Set	62	0.77	0.71	0.558(0.451-0.664)	0.32
	Cross-validation	74.69	0.77	0.71	0.568	0.40
**Female**	Test Set	68	0.79	0.67	0.702(0.606-0.797)	0.68
	Cross-validation	65.15	0.69	0.61	0.594	0.47

For peer review only - http://bmjopen.bmj.com/site/about/guidelines.xhtml

Figure Legends:

Figure 1: Overall Results: Partial dependency plots for predicting test positive results for first 10 most influential variables using permutation methods in the overall population using Shrinkage=0.01, bag fraction=0.5, interaction depth=5, CV fold=10, train fraction=0.7, n.minobsinnode = 10 as hyperparameters from grid search

Figure 2: Results for Females: Partial dependency plots for predicting test positive results for first 5 most influential variables using permutation methods in Females using Shrinkage=0.01, bag fraction=1, interaction depth=7, CV fold=10, train fraction=0.8, n.minobsinnode = 15 as hyperparameters from grid search

Figure 3: Results for Males: Partial dependency plots for predicting test positive results for first 5 most influential variables using permutation methods in males using, Shrinkage=0.001, bag fraction=1, interaction depth=3, CV fold=10, train fraction=0.8, n.minobsinnode = 5 as hyperparameters from grid search

Figure 4: Hospitalization in overall population: Partial dependency plots for predicting hospitalization in test positive patients for first 5 most influential variables using permutation methods using, Shrinkage=0.1, bag fraction=0.8, interaction depth=3, CV fold=10, train fraction=0.7, n.minobsinnode = 15 as hyperparameters from grid search

Figure 5: Hospitalization of females: Partial dependency plot for predicting hospitalization in test positive females for first 5 most influential variables using permutation methods using, Shrinkage=0.05, bag fraction=0.65, interaction depth=7, CV fold=10, train fraction=0.8, n.minobsinnode = 15 as hyperparameters from grid search

Figure 6: Hospitalization of males: Partial dependency plot for predicting hospitalized patients in test positive males for first 5 most influential variables using permutation methods using, Shrinkage=0.1, bag fraction=0.5, interaction depth=5, CV fold=10, train fraction=0.8, n.minobsinnode = 15 as hyperparameters from grid search

For peer review only - http://bmjopen.bmj.com/site/about/guidelines.xhtml

References:

1. Johns Hopkins University J. COVID-19 Dashboard by the Center for Systems Science and Engineering (CSSE) 2020 [Available from: https://www.arcgis.com/apps/opsdashboard/index.html#/bda7594740fd40299423467b48e9ecf6.
2. Niedzwiedz CL, O'Donnell CA, Jani BD, et al. Ethnic and socioeconomic differences in SARS-CoV-2 infection: prospective cohort study using UK Biobank. BMC Medicine 2020;**18**(1):1-14.
3. Lassale C, Gaye B, Hamer M, et al. Ethnic Disparities in Hospitalization for COVID-19: a Community-Based Cohort Study in the UK. medRxiv 2020.
4. Prats-Uribe A, Paredes R, Prieto-Alhambra D. Ethnicity, comorbidity, socioeconomic status, and their associations with COVID-19 infection in England: a cohort analysis of UK Biobank data. medRxiv 2020.
5. Kolin DA, Kulm S, Elemento O. Clinical and Genetic Characteristics of Covid-19 Patients from UK Biobank. medRxiv 2020.
6. Hamer M, Kivimäki M, Gale CR, et al. Lifestyle Risk Factors, Inflammatory Mechanisms, and COVID-19 Hospitalization: A Community-Based Cohort Study of 387,109 Adults in UK. Brain, Behavior, and Immunity 2020.
7. Patel AP, Paranjpe MD, Kathiresan NP, et al. Race, Socioeconomic Deprivation, and Hospitalization for COVID-19 in English participants of a National Biobank. medRxiv 2020.
8. Darling AL, Ahmadi KR, Ward KA, et al. Vitamin D status, body mass index, ethnicity and COVID-19: Initial analysis of the first-reported UK Biobank COVID-19 positive cases (n 580) compared with negative controls (n 723). medRxiv 2020.
9. Schiffer VM, Janssen EB, van Bussel BC, et al. The" sex gap" in COVID-19 trials: a scoping review. EClinicalMedicine 2020:100652.
10. Tadiri CP, Gisinger T, Kautzy-Willer A, et al. The influence of sex and gender domains on COVID-19 cases and mortality. CMAJ 2020;**192**(36):E1041-E45.
11. COVID-19 and sex differences: mechanisms and biomarkers. Mayo Clinic Proceedings; 2020. Elsevier.
12. Scully EP, Haverfield J, Ursin RL, et al. Considering how biological sex impacts immune responses and COVID-19 outcomes. Nature Reviews Immunology 2020:1-6.
13. Galbadage T, Peterson BM, Awada J, et al. Systematic Review and Meta-Analysis of Sex-Specific COVID-19 Clinical Outcomes. medRxiv 2020.
14. Johnson JL, Greaves L, Repta R. Better science with sex and gender: Facilitating the use of a sex and gender-based analysis in health research. International Journal for Equity in Health 2009;**8**(1):1-11.
15. CIHR OTM. Online Training Modules: Integrating Sex & Gender in Health Research 2020 [Available from: https://cihr-irsc.gc.ca/e/49347.html.
16. World Health Organization (WHO) S. Addressing sex and gender in epidemic-prone infectious diseases 2007 [Available from: https://www.who.int/csr/resources/publications/SexGenderInfectDis.pdf.
17. Al Youha S, Alkhamis M, Al Mazeedi S, et al. Using Machine Learning to Unveil Demographic and Clinical Features of COVID-19 Symptomatic and Asymptomatic Patients. 2020.
18. KC W, So H. Uncovering clinical risk factors and prediction of severe COVID-19: A machine learning approach based on UK Biobank data. 2020.
19. Ahamad MM, Aktar S, Rashed-Al-Mahfuz M, et al. A machine learning model to identify early stage symptoms of SARS-Cov-2 infected patients. Expert systems with applications 2020;**160**:113661.
20. Sudlow C, Gallacher J, Allen N, et al. UK biobank: an open access resource for identifying the causes of a wide range of complex diseases of middle and old age. Plos med 2015;**12**(3):e1001779.
21. Armstrong J, Rudkin JK, Allen N, et al. Dynamic linkage of covid-19 test results between public health england's second generation surveillance system and uk biobank. Microbial genomics 2020;**6**(7).
22. Mutambudzi M, Niedzwiedz CL, Macdonald EB, et al. Occupation and risk of COVID-19: prospective cohort study of 120,621 UK Biobank participants. medRxiv 2020.
23. Pilote L, Norris CM, Raparelli V, et al. Gender Outcomes INternational Group: to Further Well-being Development (GOING-FWD): https://www.mcgill.ca/going-fwd4gender/, 2020.
24. Tadiri CP, Raparelli V, Abrahamowicz M, et al. Methods for prospectively incorporating gender into health sciences research. Journal of Clinical Epidemiology 2020.

For peer review only - http://bmjopen.bmj.com/site/about/guidelines.xhtml

25. Friedman JH. Greedy function approximation: a gradient boosting machine. Annals of statistics 2001:1189-232.
26. Ridgeway G. Generalized Boosted Models: A guide to the gbm package. Update 2007;**1**(1):2007.
27. Bühlmann P, Hothorn T. Boosting algorithms: Regularization, prediction and model fitting. Statistical Science 2007;**22**(4):477-505.
28. Maleki F, Ovens K, Najafian K, et al. Overview of Machine Learning Part 1: Fundamentals and Classic Approaches. Neuroimaging Clin N Am 2020;**30**(4):e17-e32.
29. Maleki F, Muthukrishnan N, Ovens K, et al. Machine Learning Algorithm Validation: From Essentials to Advanced Applications and Implications for Regulatory Certification and Deployment. Neuroimaging Clin N Am 2020;**30**(4):433-45.
30. Yan Y. MLmetrics: Machine learning evaluation metrics. R package version 2016;**1**(1).
31. Nicodemus KK, Malley JD, Strobl C, et al. The behaviour of random forest permutation-based variable importance measures under predictor correlation. BMC bioinformatics 2010;**11**(1):1-13.
32. Greenwell BM. pdp: An R Package for Constructing Partial Dependence Plots. R J 2017;**9**(1):421.
33. Gebhard C, Regitz-Zagrosek V, Neuhauser HK, et al. Impact of sex and gender on COVID-19 outcomes in Europe. Biology of Sex Differences 2020;**11**(1):1-13.
34. Yates T, Razieh C, Zaccardi F, et al. Obesity and risk of COVID-19: analysis of UK Biobank. Primary Care Diabetes 2020.
35. Atkins JL, Masoli JA, Delgado J, et al. Preexisting comorbidities predicting severe COVID-19 in older adults in the UK Biobank community cohort. medRxiv 2020.
36. Guo W, Li M, Dong Y, et al. Diabetes is a risk factor for the progression and prognosis of COVID-19. Diabetes/metabolism research and reviews 2020:e3319.
37. Scalsky RJ, Desai K, Chen Y-J, et al. Baseline Cardiometabolic Profiles and SARS-CoV-2 Risk in the UK Biobank. medRxiv 2020.
38. Aldridge RW, Lewer D, Katikireddi SV, et al. Black, Asian and Minority Ethnic groups in England are at increased risk of death from COVID-19: indirect standardisation of NHS mortality data. Wellcome Open Research 2020;**5**(88):88.

For peer review only

For peer review only - http://bmjopen.bmj.com/site/about/guidelines.xhtml

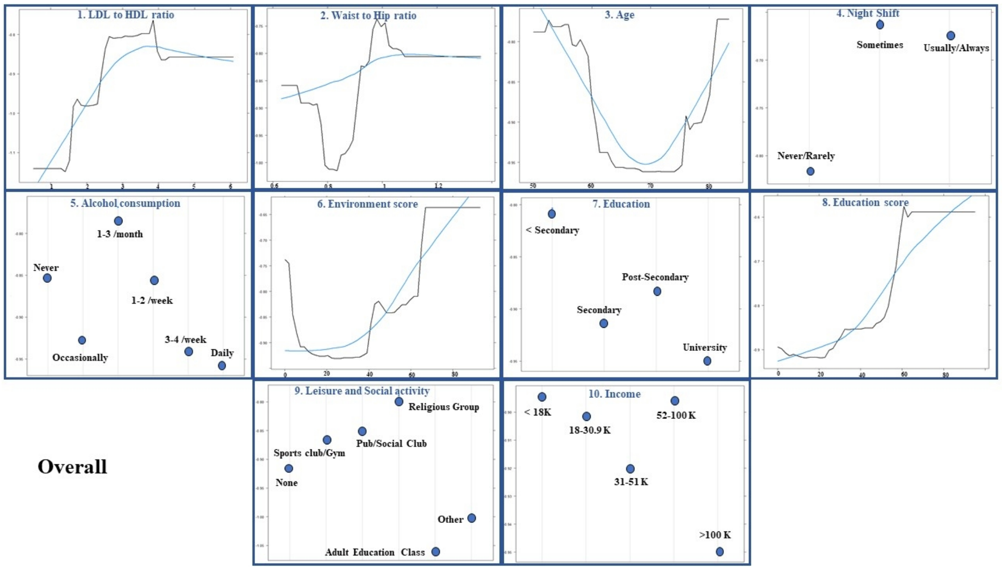

Figure 1: Overall Results: Partial dependency plots for predicting test positive results for first 10 most influential variables using permutation methods in the overall population using Shrinkage=0.01, bag fraction=0.5, interaction depth=5, CV fold=10, train fraction=0.7, n.minobsinnode = 10 as hyperparameters from grid search

338x190mm (96 x 96 DPI)

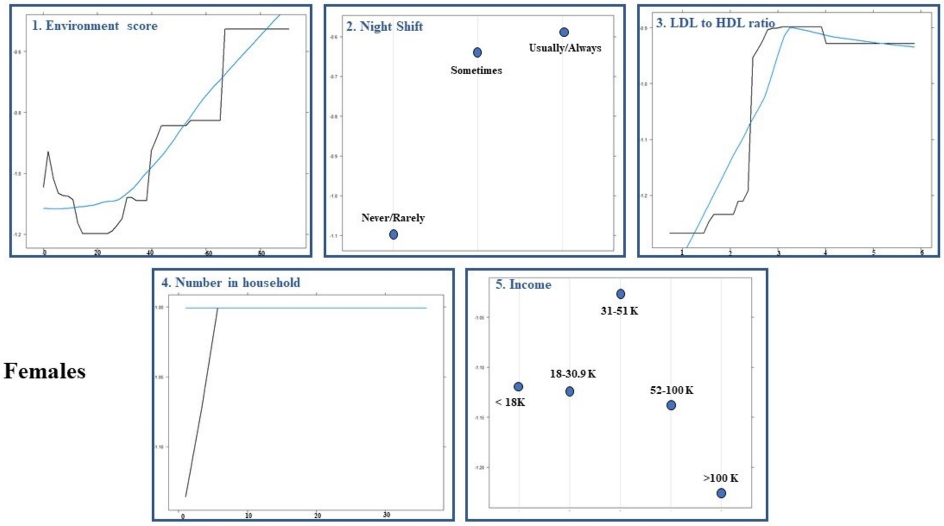

Figure 2: Results for Females: Partial dependency plots for predicting test positive results for first 5 most influential variables using permutation methods in Females using Shrinkage=0.01, bag fraction=1, interaction depth=7, CV fold=10, train fraction=0.8, n.minobsinnode = 15 as hyperparameters from grid search

306x178mm (96 x 96 DPI)

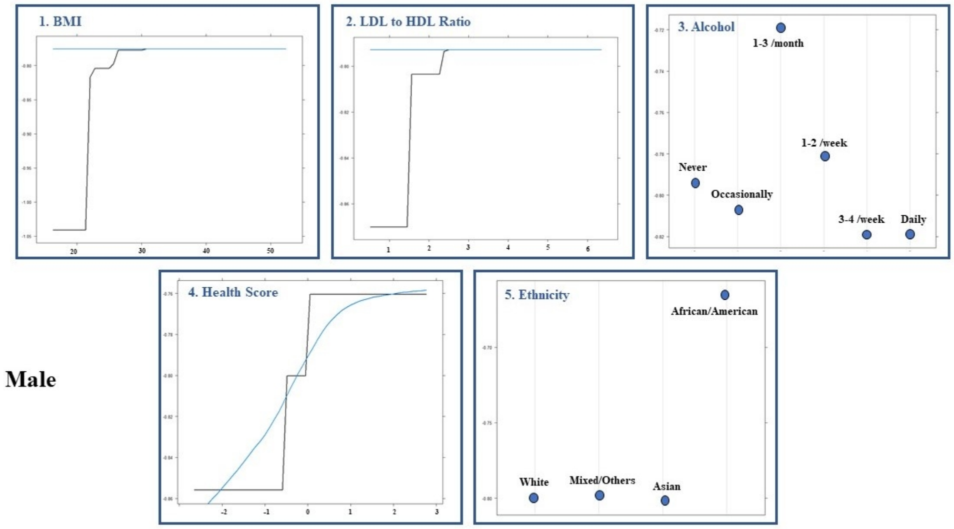

Figure 3: Results for Males: Partial dependency plots for predicting test positive results for first 5 most influential variables using permutation methods in males using, Shrinkage=0.001, bag fraction=1, interaction depth=3, CV fold=10, train fraction=0.8, n.minobsinnode = 5 as hyperparameters from grid search

305x181mm (96 x 96 DPI)

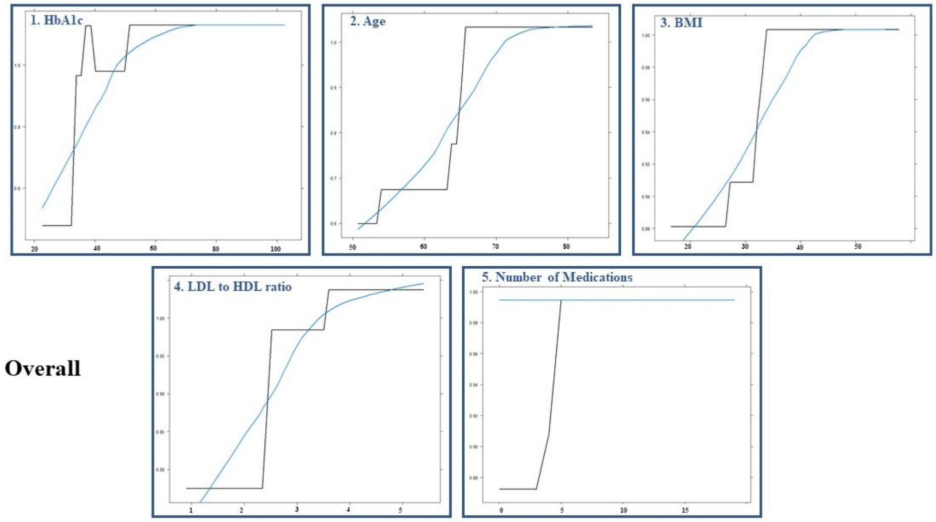

Figure 4: Hospitalization in overall population: Partial dependency plots for predicting hospitalization in test positive patients for first 5 most influential variables using permutation methods using, Shrinkage=0.1, bag fraction=0.8, interaction depth=3, CV fold=10, train fraction=0.7, n.minobsinnode = 15 as hyperparameters from grid search

309x178mm (96 x 96 DPI)

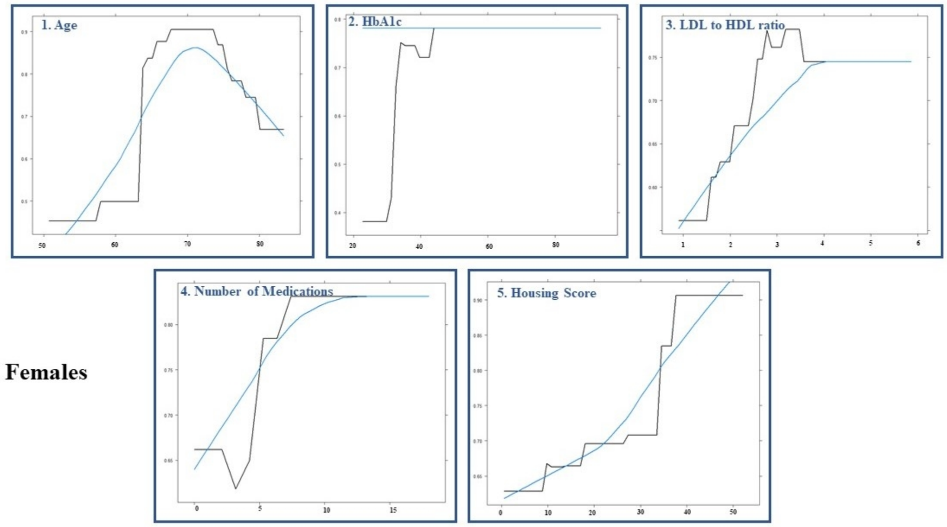

Figure 5: Hospitalization of females: Partial dependency plot for predicting hospitalization in test positive females for first 5 most influential variables using permutation methods using, Shrinkage=0.05, bag fraction=0.65, interaction depth=7, CV fold=10, train fraction=0.8, n.minobsinnode = 15 as hyperparameters from grid search

305x181mm (96 x 96 DPI)

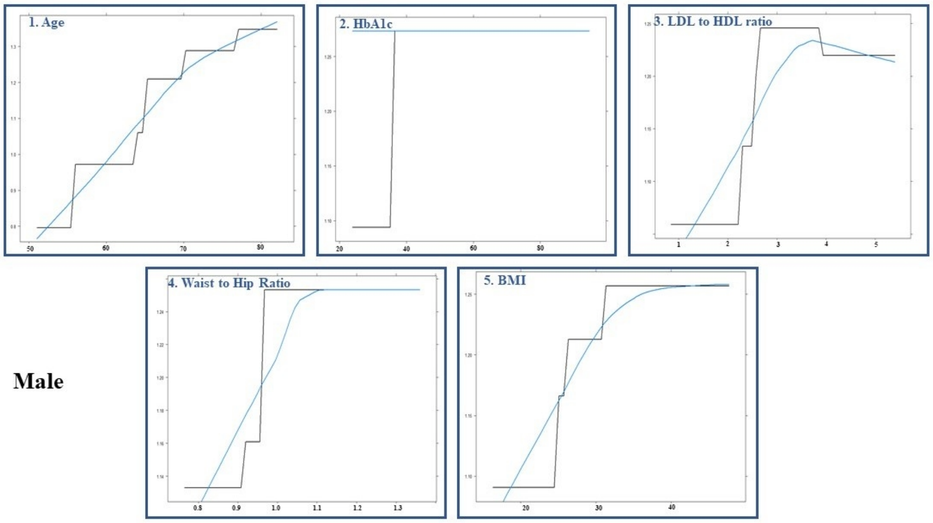

Figure 6: Hospitalization of males: Partial dependency plot for predicting hospitalized patients in test positive males for first 5 most influential variables using permutation methods using, Shrinkage=0.1, bag fraction=0.5, interaction depth=5, CV fold=10, train fraction=0.8, n.minobsinnode = 15 as hyperparameters from grid search

308x180mm (96 x 96 DPI)

Supplemental

Supplemental 1. Definition of deprivation indices

Variable Name	**Definition**
Education score	This measure shows the extent of deprivation in education, skills and training in an area within 2 sub domains including children and young people and adults skills.
Living environment score	This score measures the quality of individuals' immediate surroundings both within and outside the home within the 'indoors' living environment, which measures the quality of housing, and the 'outdoors' living environment which contains two measures relating to air quality and road traffic accidents subdomains.
Employment score	This score measures employment deprivation in an area conceptualized as involuntary exclusion of the working age population from the labour market.
Income score	This score measures the proportion of the population in an area experiencing deprivation related to low income.
Housing score	This score measures the physical and financial accessibility of housing and key local services. The indicators fall into two sub-domains: 'geographical barriers', which relate to the physical proximity of local services, and 'wider barriers' which includes issues relating to access to housing such as affordability.
Health score	This score measures premature death and the impairment of quality of life by poor health. It considers both physical and mental health. The domain measures morbidity, disability and premature mortality but not aspects of behaviour or environment that may be predictive of future health deprivation.

For peer review only - http://bmjopen.bmj.com/site/about/guidelines.xhtml

STROBE 2007 (v4) checklist of items to be included in reports of observational studies in epidemiology*

Checklist for cohort, case-control, and cross-sectional studies (combined)

Section/Topic	Item #	Recommendation	Reported on page #
**Title and abstract**	1	(*a*) Indicate the study's design with a commonly used term in the title or the abstract	1-2
		(*b*) Provide in the abstract an informative and balanced summary of what was done and what was found	1-2
**Introduction**			
Background/rationale	2	Explain the scientific background and rationale for the investigation being reported	4-5
Objectives	3	State specific objectives, including any pre-specified hypotheses	4-5
**Methods**			
Study design	4	Present key elements of study design early in the paper	5
Setting	5	Describe the setting, locations, and relevant dates, including periods of recruitment, exposure, follow-up, and data collection	5
Participants	6	(*a*) *Cohort study*—Give the eligibility criteria, and the sources and methods of selection of participants. Describe methods of follow-up<br>*Case-control study*—Give the eligibility criteria, and the sources and methods of case ascertainment and control selection. Give the rationale for the choice of cases and controls<br>*Cross-sectional study*—Give the eligibility criteria, and the sources and methods of selection of participants	5
		(*b*) *Cohort study*—For matched studies, give matching criteria and number of exposed and unexposed<br>*Case-control study*—For matched studies, give matching criteria and the number of controls per case	
Variables	7	Clearly define all outcomes, exposures, predictors, potential confounders, and effect modifiers. Give diagnostic criteria, if applicable	6-7
Data sources/ measurement	8*	For each variable of interest, give sources of data and details of methods of assessment (measurement). Describe comparability of assessment methods if there is more than one group	*6-7*
Bias	9	Describe any efforts to address potential sources of bias	NA
Study size	10	Explain how the study size was arrived at	5
Quantitative variables	11	Explain how quantitative variables were handled in the analyses. If applicable, describe which groupings were chosen and why	7
Statistical methods	12	(*a*) Describe all statistical methods, including those used to control for confounding	7-8
		(*b*) Describe any methods used to examine subgroups and interactions	7-8
		(*c*) Explain how missing data were addressed	7
		(*d*) *Cohort study*—If applicable, explain how loss to follow-up was addressed<br>*Case-control study*—If applicable, explain how matching of cases and controls was addressed	

For peer review only

		Cross-sectional study—If applicable, describe analytical methods taking account of sampling strategy	
		(*e*) Describe any sensitivity analyses	7-8
**Results**			
Participants	13*	(a) Report numbers of individuals at each stage of study—eg numbers potentially eligible, examined for eligibility, confirmed eligible, included in the study, completing follow-up, and analysed	9
		(b) Give reasons for non-participation at each stage	NA
		(c) Consider use of a flow diagram	NA
Descriptive data	14*	(a) Give characteristics of study participants (eg demographic, clinical, social) and information on exposures and potential confounders	9
		(b) Indicate number of participants with missing data for each variable of interest	-
		(c) *Cohort study*—Summarise follow-up time (eg, average and total amount)	-
Outcome data	15*	*Cohort study*—Report numbers of outcome events or summary measures over time	-
		Case-control study—Report numbers in each exposure category, or summary measures of exposure	-
		Cross-sectional study—Report numbers of outcome events or summary measures	9-10
Main results	16	(*a*) Give unadjusted estimates and, if applicable, confounder-adjusted estimates and their precision (eg, 95% confidence interval). Make clear which confounders were adjusted for and why they were included	9-10
		(*b*) Report category boundaries when continuous variables were categorized	9-10
		(*c*) If relevant, consider translating estimates of relative risk into absolute risk for a meaningful time period	9-10
Other analyses	17	Report other analyses done—eg analyses of subgroups and interactions, and sensitivity analyses	9-10
**Discussion**			
Key results	18	Summarise key results with reference to study objectives	10-11
Limitations	19	Discuss limitations of the study, taking into account sources of potential bias or imprecision. Discuss both direction and magnitude of any potential bias	13
Interpretation	20	Give a cautious overall interpretation of results considering objectives, limitations, multiplicity of analyses, results from similar studies, and other relevant evidence	14
Generalisability	21	Discuss the generalisability (external validity) of the study results	14
**Other information**			
Funding	22	Give the source of funding and the role of the funders for the present study and, if applicable, for the original study on which the present article is based	14

*Give information separately for cases and controls in case-control studies and, if applicable, for exposed and unexposed groups in cohort and cross-sectional studies.
Note: An Explanation and Elaboration article discusses each checklist item and gives methodological background and published examples of transparent reporting. The STROBE checklist is best used in conjunction with this article (freely available on the Web sites of PLoS Medicine at http://www.plosmedicine.org/, Annals of Internal Medicine at http://www.annals.org/, and Epidemiology at http://www.epidem.com/). Information on the STROBE Initiative is available at www.strobe-statement.org.

For peer review only

BMJ Open

The Importance of Sex and Gender Factors for COVID-19 Vaccination: A Sex-stratified Analysis Using Machine Learning

Journal:	*BMJ Open*
Manuscript ID	bmjopen-2021-050450.R1
Article Type:	Original research
Date Submitted by the Author:	16-Dec-2021

Complete List of Authors:	Azizi, Zahra; McGill University Health Centre<br>Shiba, Yumika; McGill University, Department of Biology<br>Alipour, Pouria; McGill University Health Centre; McGill University<br>Maleki, Farhad; McGill University Health Centre, Department of Radiology<br>Raparelli, Valeria; University of Ferrara, Department of Translational Medicine; University of Alberta, Department of Translational Medicine<br>Norris, Colleen; University of Alberta, Faculty of Nursing<br>Forghani, Reza; McGill University Health Centre, Department of Radiology<br>Pilote, Louise; McGill University Health Centre; McGill University<br>El Emam, Khaled; University of Ottawa; University of Ottawa<br>Investigators, GOING FWD ; McGill University

**Primary Subject Heading**:	Public health
Secondary Subject Heading:	Infectious diseases
Keywords:	Health policy < HEALTH SERVICES ADMINISTRATION & MANAGEMENT, Risk management < HEALTH SERVICES ADMINISTRATION & MANAGEMENT, COVID-19

SCHOLARONE™
Manuscripts

I, the Submitting Author has the right to grant and does grant on behalf of all authors of the Work (as defined in the below author licence), an exclusive licence and/or a non-exclusive licence for contributions from authors who are: i) UK Crown employees; ii) where BMJ has agreed a CC-BY licence shall apply, and/or iii) in accordance with the terms applicable for US Federal Government officers or employees acting as part of their official duties; on a worldwide, perpetual, irrevocable, royalty-free basis to BMJ Publishing Group Ltd ("BMJ") its licensees and where the relevant Journal is co-owned by BMJ to the co-owners of the Journal, to publish the Work in this journal and any other BMJ products and to exploit all rights, as set out in our licence.

The Submitting Author accepts and understands that any supply made under these terms is made by BMJ to the Submitting Author unless you are acting as an employee on behalf of your employer or a postgraduate student of an affiliated institution which is paying any applicable article publishing charge ("APC") for Open Access articles. Where the Submitting Author wishes to make the Work available on an Open Access basis (and intends to pay the relevant APC), the terms of reuse of such Open Access shall be governed by a Creative Commons licence – details of these licences and which Creative Commons licence will apply to this Work are set out in our licence referred to above.

Other than as permitted in any relevant BMJ Author's Self Archiving Policies, I confirm this Work has not been accepted for publication elsewhere, is not being considered for publication elsewhere and does not duplicate material already published. I confirm all authors consent to publication of this Work and authorise the granting of this licence.

For peer review only

The Importance of Sex and Gender Factors for COVID-19 Vaccination: A Sex-stratified Analysis Using Machine Learning

Zahra Azizi, MD, MSc [1]; Yumika Shiba [2]; Pouria Alipour, MSc[1,3]; Farhad Maleki, PhD[4]; Valeria Raparelli, MD, PhD [5,6]; Colleen Norris, PhD [6,7]; Reza Forghani, MD, PhD[4]; Louise Pilote, MD, PhD* [1,3,8]; Khaled El-Emam, PhD* [9,10,11] and the GOING FWD Investigators

***Drs Pilote and El-Emam are co-senior authors**

1. Centre for Outcomes Research and Evaluation, McGill University Health Centre Research Institute, Montreal, QC, Canada
2. Department of Biology; McGill University, Montreal, QC, Canada
3. Faculty of Medicine and Health Sciences, McGill University, Montreal, QC, Canada
4. Augmented Intelligence & Precision Health Laboratory (AIPHL), Department of Radiology, McGill University Health Centre, Montreal, QC, Canada
5. Department of Translational Medicine, University of Ferrara, Ferrara, Italy
6. Faculty of Nursing, University of Alberta, Edmonton, Alberta, Canada
7. Heart and Stroke Strategic Clinical Networks-Alberta Health Services, Alberta, Canada
8. Divisions of Clinical Epidemiology and General Internal Medicine, McGill University Health Centre Research Institute, Montreal, QC, Canada
9. Children's Hospital of Eastern Ontario Research Institute, Ottawa, Ontario, Canada
10. Faculty of Medicine, University of Ottawa, Ottawa, Ontario. Canada
11. Replica Analytics Ltd, Ottawa, Ontario, Canada

Corresponding Authors:

Louise Pilote MD, MPH, PhD
Center for Outcomes Research and Evaluation
Divisions of Clinical Epidemiology and General Internal Medicine
McGill University Health Centre Research Institute
5252 boulevard de Maisonneuve, Montreal
Quebec, H3A 1A1, Canada
Tel: 514 934-1934 ext. 44722; Fax: 514 843-1676
E-mail: louise.pilote@mcgill.ca

Khaled El Emam, PhD
Children's Hospital of Eastern Ontario Research Institute
401 Smyth Road, Ottawa
Ontario K1J 8L1, Canada
E-mail: kelemam@ehealthinformation.ca
Tel: +1 613-797-5412

Word Count: 3781 (w/o abstract and references)

For peer review only - http://bmjopen.bmj.com/site/about/guidelines.xhtml

Abstract:

Objective: To examine sex and gender roles in COVID-19 test positivity and hospitalization in sex-stratified predictive models using machine learning.

Design: Cross-sectional study

Setting: UK Biobank prospective cohort

Participants: Participants tested between March 16th, 2020, to May 18th, 2020, were analyzed.

Main outcome measures: The endpoints of the study were COVID-19 test positivity and hospitalization. Forty-two individuals' demographics, psychosocial factors, and comorbidities were used as likely determinants of outcomes. Gradient boosting machine was utilized for building prediction models.

Results: Of 4,510 individuals tested (51.2% females, mean age= 68.5±8.9 years), 29.4% tested positive. Males were more likely to be positive than females (31.6% vs 27.3%, P=0.001). In females, living in more deprived areas, lower income, increased LDL-to-HDL ratio, working night shifts, and living with a greater number of family members were associated with a higher likelihood of COVID-19 positive test. While in males, greater body mass index and LDL-to-HDL ratio were the factors associated with a positive test. Older age, and adverse cardio-metabolic characteristics were the most prominent variables associated with hospitalization of test positive patients in both overall, and sex-stratified models.

Conclusion: High-risk jobs, crowded living arrangements, and living in deprived areas were associated with increased COVID-19 infection in females, while high-risk cardio-metabolic characteristics were more influential in males. Gender-related factors have a greater impact in females; hence they should be considered in identifying priority groups for COVID-19 infection vaccination campaigns.

Keywords: Covid-19 infection, SARS-Cov-2, Test Positive, Hospitalization, Sex, Gender, Machine

For peer review only - http://bmjopen.bmj.com/site/about/guidelines.xhtml

Learning, Vaccination

Strengths and limitations:

- A unique feature of the study is the investigation of numerous psycho-socio-cultural factors in conjunction with clinical and laboratory factors made feasible through machine learning algorithms.
- The assessment of sex-stratified algorithms to elucidate the most influential factors in both sexes adds novel information as it has rarely been done to date.
- The first limitation of this study is selection bias due to the lack of systematic and random testing across the UK.
- This analysis was done using the baseline diagnostic data that was collected from 2006 to 2010; therefore, misclassification of determinants is possible. However, previous studies of the UK Biobank have shown a high correlation between baseline and follow-up data for a subsample of patients who had further visits for imaging.
- Finally, the relatively low predictive model performance (Test positivity: AUC: 0.570 (95% Confidence Interval (CI): 0.537-0.604), hospitalization: AUC: 0.60 (95% CI: 0.534-0.665)) —as expected—reflects other influences not captured in the UK Biobank.

For peer review only - http://bmjopen.bmj.com/site/about/guidelines.xhtml

Introduction

The novel coronavirus SARS-CoV-2 (COVID-19) pandemic has led to more than 250 million reported positive cases and over five million deaths worldwide as of November 2021[1]. As vaccination is rolling out, continuous efforts are made to establish risk factors for the disease and find vulnerable populations across the globe [2-8].

There has been an imbalance in infection susceptibility, severity, and mortality between males and females [9]. These differences are multifactorial and have been attributed to a combination of biological (i.e., genetic, hormonal) and psycho-socio-cultural differences (gender) [9-13]. While "sex" refers to a set of biological attributes in humans and animals, "gender" refers to the roles, behaviours, and identities of individuals that forms throughout life [14 15]. It is increasingly recognized that both sex and gender play significant roles in health outcomes [14 15], including the acquisition of infections and response to infection and treatments [16]. The world health organization statement [16], addressing sex and gender in epidemic-prone infectious diseases, outlines that differences between males and females can lead to differences in activity patterns in work and in family roles, which may increase the risk of exposure to infectious disease in a particular setting [16]. Therefore, lack of consideration of the influences of sex and gender in COVID-19 contraction can potentially hinder the effectiveness of COVID-19 vaccination prioritization strategies.

With the introduction of the COVID-19 vaccines, identifying those most vulnerable to infection with accurate prediction models that account for more complex relationships is urgent. Machine learning algorithms can explore non-linear and complex relationships by considering the interaction between both biological and psycho-socio-cultural factors together; however, these methods are still underutilized in the medical field, and few predictive models have been tailored to each sex [17-19]. Therefore, we examined sex and gender-related factors associated with SARS-

For peer review only - http://bmjopen.bmj.com/site/about/guidelines.xhtml

CoV-2 test positivity and COVID-19 hospitalization in the UK Biobank cohort and developed sex-stratified predictive models using machine learning methods.

Methods

Study Population

This is a cross sectional study of UK Biobank data. The UK Biobank (UKB, https://www.ukbiobank.ac.uk/) is a prospective cohort study that collects health, lifestyle, genetic, and imaging data for over 500,000 randomly selected participants in the UK [20]. Baseline data collection took place between 2006–2010 across England, Scotland, and Wales. The age of participants at recruitment was between 40 and 69 years [20]. Data was collected in 22 Assessment Centers through 4 main methods; touchscreen questionnaires, verbal interviews, physical measures, and biological sampling [20]. For this study, only data from England between March 16th, 2020, to May 18th, 2020, was used due to the unavailability of COVID-19 test results from Scotland and Wales at the time of analysis.

Ethics approval for the project was obtained from the coordinator center at McGill University, Canada (CIHR GNP-161904, 2018/10/01 to 2023/03/31). The data was obtained from the UK Biobank repository (Application ID:45551).

Patient and Public Involvement

No patient involved in the design, or conduct, or reporting, or dissemination plans of our research.

COVID-19 Test Results

COVID-19 test results were available from Public Health England (PHE) data for 4510 participants , which was linked to UK Biobank baseline data [21]. The primary endpoint of the study

For peer review only - http://bmjopen.bmj.com/site/about/guidelines.xhtml

was test positivity. We defined testing positive as having at least one positive test result. The test results were available from March 16th, 2020, to May 18th, 2020. The secondary endpoint of the study was being hospitalized for a COVID-19 related illness. For this purpose, we chose test-positive patients who had at least one positive result in an in-patient setting. The results are from samples taken from the combined nose, throat, sputum, or lower respiratory tract. The analysis for infection was done using real-time polymerase chain reaction (RT-PCR) tests for SARS-CoV-2.

Baseline Characteristics

Patients' demographics, psychosocial (gender-related variables), anthropometric variables, and comorbidities that were collected at baseline 2006-2010 were used for analysis. We used this particular baseline data for socioeconomic status and occupation since studies have shown a high correlation between baseline and follow-up data for a subsample of patients who had further visits for imaging [22].

Gender-related Psychosocial Variables

A multistep approach for identifying gender-related variables was exploited based on GOING-FWD (Gender Outcomes International Group to Further Well-being Development) methodology [23], the Women Health Research Network's gender framework (i.e. gender identity, gender roles, gender relationships, and institutionalized gender), and the Canadian Institutes of Health Research sex and gender modules [14 15 24]. Based on this approach and data availability, the following variables were selected: multiple deprivation indices (Supplemental 1), employment, jobs involving night shift, education level, smoking status, alcohol consumption, number of children, household crowding, housing ownership, income, leisure and social activities, risk-taking

For peer review only - http://bmjopen.bmj.com/site/about/guidelines.xhtml

behaviors, and neuroticism score. Details of the variables are available in the UK Biobank data dictionary (https://biobank.ctsu.ox.ac.uk/crystal/search.cgi).

Comorbidities

Baseline comorbidities were self-reported and collected using a touch screen device—i.e., hypertension (HTN), diabetes (DM), chronic obstructive pulmonary disease (COPD), asthma, allergy, history of stroke, heart attack, angina, deep vein thrombosis (DVT), pulmonary embolism (PE), and cancer (CA). In addition, the number of medications and long-standing illness, disability, or infirmity were also included and coded as dichotomous variables. These variables were selected based on their significance as demonstrated by a number of previous investigations on the UK Biobank [2 3 5-8 18 22 25-27].

Physical and Biological Characteristics

Variables used as measures of physical and biological characteristics were body mass index (BMI) (defined as weight (Kg)/height (m)^2), waist to hip ratio (WHR), levels of vitamin D, hemoglobin A1C (HbA1C), high-density lipoprotein (HDL) and low-density lipoprotein (LDL); all coded as continuous variables.

Statistical Analyses

Descriptive statistics were reported as mean and standard deviation for continuous variables and frequency and percentage for categorical variables. Group-based differences (test negative versus positive) in baseline characteristics were compared using independent Student's t-test for continuous variables (or its non-parametric counterpart for skewed distributions) and chi-square for categorical variables. P-values of less-than or equal-to 0.05 were considered statistically significant. A complete case analysis (pairwise deletion) approach was used for dealing with missing data in the descriptive analysis. Bonferroni type adjustments were utilized to correct for

For peer review only - http://bmjopen.bmj.com/site/about/guidelines.xhtml

multiple testing and the results after adjustment were used for interpretation. Data analysis was performed using R-Studio (Version 1.3.1093) and R software (v4.0.3).

Machine Learning

The data was split into 70% training and 30% test sets, where the test set was only used for the evaluation of the final models. The training set was used to develop gradient boosting decision tree models [28-30] using the "gbm3" R package for predicting SARS-Cov-2 test-positivity and hospitalization prediction. To reduce the effect of class imbalance on model development [31], a bootstrap oversampling approach was used. For each endpoint, i.e., test-positivity or hospitalization, three different models were developed: (1) a model for males, (2) a model for females, and (3) an overall model for males and females combined. Calibration was then performed using isotonic regression method.

Using k-fold cross-validation is considered a best practice in developing machine learning models [32]. Therefore, for developing each model, a 10-fold cross-validation was performed on the training set. We also used a grid-search procedure to find the best combination of boosting hyper-parameters and tree specific hyper-parameters (e.g., learning rate (shrinkage), tree depth, bag fraction, and the minimum number of observations in terminal nodes). A Bernoulli distribution was used for classification models. These resulted in three trained models for SARS-Cov-2 test-positivity and three models for SARS-Cov-2 hospitalization.

To provide an unbiased estimate of the model generalization errors, the performance of the trained models was assessed and reported on the test set. Confusion matrix derived metrics including accuracy, precision, recall (sensitivity), specificity as well as AUC score were used as the performance measures [33]. We used the best threshold of ROC as cut off for determining precision/recall, sensitivity/specificity. Another metric which focuses on predictions of the

For peer review only - http://bmjopen.bmj.com/site/about/guidelines.xhtml

positive class is the Area Under the Precision-Recall Curve (AUPRC) [34]. Interpretation of AUPRC is dependent on the class distribution of the outcome as the minimal achievable value is dependent on that distribution [35], and the AUPRC value of a random classifier is the rate of the positive class [35].

Most Influential Variables

For identifying the most influential variables, permutation-based feature importance was utilized [36]. This approach measures a feature (variable) importance by calculating the increase of the model's prediction error after permuting the feature.

We reported partial dependence plots (PDPs) using the "pdp" package in R to understand the marginal effect of a feature on the predicted outcome. PDP demonstrates how the response variable changes as we change the value of a feature while taking into account the average effect of all the other features in the model [37]. The Y axis shows how the predicted value changes with change in predictor variables. If the line in the plot is constant near zero it means that the variable has no effect on the model. A negative value means that a specific value of the predictor variable is less likely to predict the correct class of outcome, whereas a positive value means the predictor variable has a positive impact on predicting the correct class [38]. A LOESS (locally estimated scatterplot smoothing) line is fit to show the trend.

Results

Of 4,510 patients (51.2% females, and 68.5±8.88 years) who were tested, 29.4% were positive. Females were less likely to be positive (males: 31.6% versus females: 27.3%, p=0.001). In descriptive analyses, there was a difference in age between test-positive and test-negative individuals (p<0.001); specifically, those younger than 60 years (Test positive vs negative: 28%

For peer review only - http://bmjopen.bmj.com/site/about/guidelines.xhtml

vs 21.8%) and those older than 80 years (Test + vs -: 6.2% vs 5.7%). Similarly, there was significant difference in test positivity amongst ethnicities (minority ethnicity: Test + vs -: 13.2% vs 7.6% $p<0.001$), and individuals born outside of the UK (Test + vs -: 14.3% vs 9.8%, $p<0.001$). Moreover, infected patients had a higher BMI (Test + vs - : 28.9±5.4 vs 28.2±5.4, $p<0.001$) ,WHR (Test + vs - : 0.89±0.09 vs 0.88±0.09), HbA1C (Test + vs - : 37.8±9.1 vs 37.1±8.3, p=0.03), LDL to HDL ratio (Test + vs - : 2.7±0.8 vs 2.5±0.9, $p<0.001$) , and lower HDL (Test + vs - : 1.3±0.3 vs 1.4±0.3, $p<0.001$) levels. On the other hand, smokers (Test + vs - : 11% vs 14%,P=0.02), alcohol drinkers (Test + vs - : 15.8% vs 20.8%, $p<0.001$), more educated individuals (Test + vs - : 31.2% vs 35.9%, $p<0.001$), house owners (Test + vs - : 79.6% vs 82.2%, p=0.04), and those who did not participate in any social activity (Test + vs - : 31.8% vs 32.7%, p=0.003) had a lower rate of infection. Test positive patients tended to have a greater number of people in their household (Test + vs -: 2.5±1.3 vs 2.3±1.5, $p<0.001$) and live in more deprived areas ($p<0.001$). These results held true in a sex-stratified analysis (Tables 1 and 2).

Machine Learning-based Prediction Models for SARS-Cov-2 Positive Test

The area under curve (AUC) for test positivity in the overall model, male and female specific models were 0.570 (95% confidence interval (CI): 0.537-0.604), 0.575 (0.529-0.621), 0.561 (95% CI: 0.512-0.609), respectively. The performance of the gradient boosted decision tree models is summarized in Table 3. Figure 1-3 illustrate the order of variable importance and partial dependence plots used for interpreting the results and direction of each variable in the models. The prediction models for the overall study population suggest that an increased LDL to HDL ratio, WHR, and age were associated with a higher likelihood of test positivity. Additionally, individuals who worked night shifts or lived in a more deprived area (lower environment and education scores) as well as those participating in social activities—including sports clubs, bars, and religious

For peer review only - http://bmjopen.bmj.com/site/about/guidelines.xhtml

groups—had a higher risk of having a positive test. In contrast, individuals with higher education levels, higher income, and those with daily or almost daily alcohol consumption were less likely to have a positive result.

The sex-specific models for test positivity showed that gender factors were more important in females, whereas in males, biological factors were significant contributors to test positivity. Females who lived in more deprived areas (increased environment score), had increased LDL to HDL ratio, worked night shifts, and had a greater number of family members in their household were more likely to test positive. Moreover, those with income greater than 100K were less likely to test positive. (Figure 2). In comparison, males with greater BMI and LDL to HDL ratio, more deprived area (greater score) and Black British ethnicity were more likely to test positive (Figure 3).

ML-based Prediction Models for COVID-19 Related Hospitalizations

The area under curve (AUC) for hospitalization in test positive patients in overall, male, and female specific models were 0.60 (95% CI: 0.534-0.665)), 0.544 (95% CI: 0.453-0.635), and 0.612 (95% CI: 0.532-0.692), respectively. The performance of the gradient boosted decision tree models is summarized in Table 4. Amongst the 1,326 test-positive patients, 932 (70.3%) were hospitalized (females: 413 (44.3%)). Figure 4-6 illustrate the order of variable importance and partial dependence plots used for interpreting the results and direction of each variable in the models. The result of the overall model to predict hospitalization in test-positive patients revealed that those with higher HbA1c level, older age, greater BMI, higher LDL to HDL ratio and greater number of medications had greater risk of being hospitalized (Figure 4).

The sex stratified model revealed that older age, a higher level of HbA1c, LDL to HDL ratio, a greater number of medications and higher-housing score (showing more deprived areas)

For peer review only - http://bmjopen.bmj.com/site/about/guidelines.xhtml

were most influential variables in predicting hospitalization in test-positive females (Figure 5), whereas older age, an increased HbA1c level, WHR, LDL to HDL ratio and BMI were the most influential variables associated with hospitalization in test-positive males (Figure 6).

Discussion

The present evaluation of individuals tested for SARS-CoV-2 in a UK cohort demonstrates the importance of gender-related factors along with clinical characteristics in predicting COVID-19 test positivity, hence providing guidance for identifying vaccination priority groups in the general population. Whilst factors related to the gender role of individuals were the most influential determinants in females, cardio-metabolic risk factors played a key role in males. Such a sex-specific cluster of factors associated with adverse outcomes was attenuated when considering the rate of COVID-19 related hospitalization among test-positive individuals. Notably, older age and cardio-metabolic diseases, including diabetes, obesity, and dyslipidemia, were most influential regardless of sex.

Emerging evidence has shown sex differences in contracting and severity of the infection. While most investigations have focused on biological factors as the potential culprit, few have incorporated gender determinants. Various modulating mechanisms have been suggested, including genetic factors (hormone-regulated expression of genes), the difference in innate and adaptive immune responses, as well as gendered factors such as lifestyle, behavioral and psychosocial factors [11 39 13].

Our findings reinforce and advance the current evidence related to the impact of metabolic comorbidities and older age in the COVID-19 pandemic. Obesity has been linked with impaired pulmonary function and suppression of immune response and has been recognized as

For peer review only - http://bmjopen.bmj.com/site/about/guidelines.xhtml

one of the most important factors in contracting COVID-19 infection, the severity of the disease, and mortality [4 40]. Higher BMI and WHR are some of the more influential measures that have shown a dose-response relationship with test positivity and disease severity [4 6 40]. A study on the UK Biobank data demonstrated a more than 50 percent increase in COVID-19 infection in obese and severely obese patients compared to non-obese individuals [40]. Investigation of different databases in various countries have also reported older age and comorbidities as the most important factors associated with clinical severity and mortality [41]. Specifically, diabetes has been associated with more severe disease manifestation [42]. This is consistent with our results, which demonstrated HbA1c level as one of the most prominent factors for hospitalization in the overall, and sex-stratified models. Preliminary studies have demonstrated the association between HDL level, infection, and disease severity. While the mechanism of this correlation is unknown, this can be due to the antioxidant, antithrombotic and anti-inflammatory role of HDL cholesterol [43].

A major and novel contribution of this study is the application of a sex and gender-oriented lens to inform the understanding of COVID-19 infection by conducting sex-disaggregated analyses and incorporating both sex and gender-related determinants. Studies have shown socioeconomic disadvantages such as living in a more deprived area and lower education to be associated with increased risk of infection and disease severity [2 4]; these factors prevail in individuals more likely to work in service-based occupations, be self-employed, or live in crowded households [3 4 44]. Moreover, less access to healthcare is another factor that leads to greater infection risk and worst outcomes. By the same token, our study demonstrated that those who live in a more deprived area with lower SES and education were more susceptible to COVID-19 infection, but the impact of gender determinants was more significant amongst

For peer review only - http://bmjopen.bmj.com/site/about/guidelines.xhtml

females. Women's role as caregivers within family and society increases their risk of infection [39]. Moreover, women are more likely to work as frontline workers, including nursing positions, increasing their exposure to the virus [3 22 39]. In our current study, environment score, household arrangement, working night shifts, and income were amongst the most important factors for females. In contrast, obesity, LDL to HDL ratio, and alcohol consumption were amongst the most influential factors in men.

The results of this study serve as an important guide for vaccination prioritization policies. While essential workers and elderly individuals have already been targeted for vaccination, the next step will be the identification and prompt vaccination of individuals in higher-risk groups in the general population. Although factors such as diabetes and obesity might be important, psychosocial risk factors such as lower socioeconomic status, education level, living in a more deprived environment, risky occupation, and household crowding should also be taken into account. Individuals, especially females, exhibiting such high-risk gendered factors should be prioritized for vaccination.

Strengths and Limitations

A unique feature of the study is the investigation of numerous lifestyles, socioeconomic, mental, and behavioral factors representing different dimensions of gender in conjunction with clinical and laboratory factors made feasible through machine learning algorithms. Furthermore, the assessment of sex-stratified algorithms to elucidate the most influential factors in both sexes adds novel information as it has rarely been done to date.

The study should also be interpreted considering some limitations. The first limitation of this study is selection bias due to the lack of systematic and random testing across the UK. Second, this analysis was done using the baseline data that was collected from 2006 to 2010 and

For peer review only - http://bmjopen.bmj.com/site/about/guidelines.xhtml

not the ones done at the time of COVID infection diagnosis. Therefore, misclassification of determinants is possible. However, previous studies of the UK Biobank have shown a high correlation between baseline and follow-up data for a subsample of patients who had further visits for imaging [22]. Moreover, a disproportionately higher representation of Caucasians in the study may make the results less generalizable to other ethnic groups. Finally, though we attempted to test various clinical, social and demographic factors to predict test positivity, it is essential to note that acquisition of infection is a multifactorial phenomenon that cannot be easily encoded using a small set of variables. Our relatively low predictive model performance—as expected—reflects other influences not captured in the UK Biobank. Similar results were obtained using the XGboost method from a recent study on the UK Biobank dataset whereby slightly superior performance to our gbm model was obtained, which further supports the interpretation that this is the expected accuracy for predictive models on this dataset[18]. While, similar features were obtained for predicting severity (hospitalization and fatality) in this study, combining mortality with hospitalization for assessing severity can justify better model performance compared to only hospitalization in our models. The difference observed in the performance of our model compared to the aforementioned study maybe also explained by the lower power and heterogeneity in our sample. Moreover, since the predictive accuracy of the model is slightly low, the risk factors deduced may not be strong enough to predict the outcomes. Additionally with the emergence of the dominant delta variant further studies are needed to elucidate the risk factors, though we suspect them to remain the same. Finally, the small sample size for this analysis may limit generalizability.

Conclusions

Sex-specific risk patterns of COVID-19 test-positivity exist, with gender-related factors

For peer review only - http://bmjopen.bmj.com/site/about/guidelines.xhtml

being more relevant in females and biological factors in males. Specifically, socioeconomic status, education level, number of people living in a household, and high-risk jobs were associated with a higher likelihood of contracting the infection in female individuals, whereas cardiometabolic disease and obesity were more associated in males. The rate of COVID-19 related hospitalization recognized similar favoring clinical factors regardless of sex. This study highlights the importance of prioritizing high-risk groups using psychosocial determinants along with clinical factors as a targeted approach for vaccination of more at-risk population to contain SARS-Cov-2 pandemic.

Funding: The GOING-FWD Consortium is funded by the GENDER-NET Plus ERA-NET Initiative (Project Ref. Number: GNP-78): The Canadian Institutes of Health Research (GNP-161904), "La Caixa" Foundation (ID 100010434) with code LCF/PR/DE18/52010001, The Swedish Research Council (2018-00932) and The Austrian Science Fund (FWF, I 4209). VR was funded by the Scientific Independence of Young Researcher Program of the Italian Ministry of University, Education, and Research (RBSI14HNVT).

Contributors:

Drs Pilote and El-Emam contributed equally as co-senior authors. ZA, YS, PA, CN, VR, LP, KEE and GOING FWD Investigators designed the study, developed the questions and contributed to the development of conceptual framework and writing the first draft. ZA, YS, and KEE performed the main analysis and critical revisions of manuscript. FM and RF validated the results by performing secondary analyses and finalizing the draft. ZA, YS, PA, CN, VR, LP, KEE and GOING FWD Investigators supervised the project from study design to final draft and approved the final version of manuscript. All Authors agree to be accountable for all aspects of the work ensuring that questions related to the accuracy or integrity of any part of the work are appropriately investigated and resolved.

For peer review only - http://bmjopen.bmj.com/site/about/guidelines.xhtml

Disclosures:

The authors disclose no conflict of interest related to this study.

Table 1. Descriptive characteristics of COVID-19 test positive versus negative males and females

**Baseline Characteristics**	**Overall**				**Females**			**Males**		
	**All**	**Test -**	**Test +**	**P**	**Test -**	**Test +**	**P**	**Test -**	**Test +**	**P**
	**N=4510**	**N=3184**	**N=1326**		**N=1679**	**N=630**		**N=1505**	**N=696**	
**Demographics**										
**Age Group, %**										
<60 years	23.6	21.8	28	<0.001*	26.4	34.1	0.001*	16.5	22.4	0.006
>=60 - <70	24.7	25.4	23		26.6	25.6		24	20.7	
>=70 -<80	45.9	47.1	42.8		42	35.1		52.9	49.9	
>=80	5.9	5.7	6.2		4.9	5.2		6.6	7	
**Sex, %**										
Female	51.2	52.7	47.5	0.001*						
**Ethnicity, %**										
White	90.7	92.4	86.8	<0.001*	91.5	86.4	<0.001*	93.4	87.1	<0.001*
Mixed	2.3	2.2	2.4		2.6	2.2		1.7	2.6	
Asian	3.3	2.6	5		2.1	5.3		3.1	4.8	
Black British	3.7	2.9	5.8		3.8	6.1		1.9	5.5	
**Country of Birth, %**										
United Kingdom	88.9	90.2	85.7	<0.001*	89.1	84	0.001*	91.5	87.4	0.003
**Biological Variables**										
**Number of Medications**										
	3.3±3.3	3.3±3.3	3.2±3.3	0.7	3.2±3.2	3.2±3.3	0.8	3.3±3.4	3.3±3.3	0.6
**BMI (Kg/m2)**										
	28.4±5.4	28.2±5.4	28.9±5.4	<0.001*	28.1±5.8	28.7±6.2	0.02	28.3±4.9	29.1±4.7	<0.001*
**WHR**										
	0.9±0.1	0.8±0.09	0.8±0.09	<0.001*	0.8±0.07	0.8±0.07	0.03	0.9	0.9	0.1
**HTN, %**										
	35.3	34.5	37	0.1	28.8	31.7	0.1	41	41.7	0.7
**Stroke, %**										
	3	3	3	1	1.7	2.1	0.6	4.4	3.8	0.5
**Heart Attack, %**										
	4	4.1	3.8	0.7	1.4	1.8	0.6	7.1	5.7	0.2
**Angina, %**										
	5.7	5.9	5.3	0.4	3.3	3.4	1	8.8	7.1	0.2
**DVT, %**										
	3.3	3.1	4	0.1	2.9	4.1	0.1	3.3	3.8	0.6
**PE, %**										
	1.7	1.9	1.1	0.09	1.9	1.4	0.6	1.9	0.9	0.09
**COPD, %**										
	3.2	3.2	3.1	0.9	2.6	2.6	1	3.9	3.6	0.8
**Asthma, %**										
	13.5	13.6	13	0.5	16.4	16.3	0.9	10.5	10	0.7
**Allergy, %**										
	21.9	22.8	19.8	0.03	26.2	22.6	0.08	19	17.3	0.3
**Diabetes, %**										

For peer review only - http://bmjopen.bmj.com/site/about/guidelines.xhtml

	9.8	9.6	10.3	0.5	7.4	8	0.7	12	12.4	0.8
**Cancer, %**										
	9.4	10.1	7.7	0.01	11.1	9.2	0.2	9.1	6.4	0.4
**Disability, %**										
	45.6	46.1	44.3	0.2	42.2	38.9	0.1	50.3	49.2	0.6
**Vitamin D level (nmol/L)**										
	46.5±21.2	46.6±21	46.1±21.6	0.4	45.7±20.7	45.2±20.9	0.6	47.6±21.4	46.7±22.2	0.4
**HbA1C level (mmol/mol)**										
	37.3±8.5	37.1±8.3	37.8±9.1	0.03	36.6±7.7	36.7±8.1	0.7	37.7±8.9	38.7±9.8	0.02
**LDL level (mmol/l)**										
	3.4±0.8	3.4±0.8	3.4±0.8	0.7	3.4±0.8	3.5±0.8	0.5	3.3±0.8	3.3±0.8	0.8
**HDL level (mmol/l)**										
	1.4±0.3	1.4±0.3	1.3±0.3	<0.001*	1.5±0.3	1.4±0.3	<0.001*	1.3±0.3	1.2±0.2	0.004
**LDL to HDL Ratio**										
	2.6±0.9	2.5±0.9	2.7±0.8	<0.001*	2.3±0.8	2.5±0.8	<0.001*	2.8±0.9	2.8±0.8	0.3

Results are presented as mean ± standard deviation for numerical variables and percentage for categorical variables.
BMI: Body Mass Index, WHR: Waist to Hip Ratio, HTN: Hypertension, DVT: Deep Vein Thrombosis, PE: Pulmonary Embolism, COPD: Chronic Obstructive Pulmonary Disease, HbA1c: Hemoglobin A1c, LDL: Low Density Lipoprotein, HDL: High Density Lipoprotein
****Bonferroni Correction=0.05/24 (number of variables) =0.002, Based on this correction the differences marked with * are considered statistically significant***

For peer review only - http://bmjopen.bmj.com/site/about/guidelines.xhtml

Table 2. Gender variables in patients who tested positive compared to test-negative patients in overall, males and females

Gender Variables,	Overall				Female			Male		
	All N=4474	Test - N=3161	Test + N=1313	P	Test - N=1668	Test + N=625	P	Test - N=1493	Test + N=688	P
**Behavioral/Lifestyle Risk Factors**										
**Smoking, %**										
0=Never	48.5	48.3	49	0.02	54.5	58.7	0.1	41.3	40.1	<0.001*
1=Previous	38.4	37.8	40		34.5	30.9		41.5	48.3	
2=Current	13.1	14	11		11	10.4		17.2	11.6	
**Alcohol, %**										
0=never	11.6	10.9	13.3	<0.001*	11.8	16.4	0.001*	9.9	10.6	0.002*
1=occasionally	14.1	14.1	14.2		18.4	18.3		9.3	10.4	
2=1 o 3 times/month	11.8	11	13.9		14	16.9		7.7	11.1	
3=1-2/week	24.1	23.6	25.4		24.1	23.8		23.1	26.8	
4=3-4/week	19	19.6	17.4		16.2	14.1		23.4	20.4	
5=daily/almost daily	19.4	20.8	15.8		15.6	10.5		26.7	20.7	
**Gender Related Variables**										
**Employment Status, %**										
Employed	50.4	49.6	52.4	0.09	53.7	58.6	0.04	45	46.7	0.5
**Night Shift, %**										
0: Never/rarely	45.1	47.5	41.4	0.21	49	40.9	0.17	45.6	42	0.8
1: Sometimes	32.1	31.6	33		31.5	32.5		31.7	33.6	
2: Usually/Always	22.8	20.9	25.6		19.5	26.6		22.8	24.4	
**Number of Children**										
	1.9±1.4	1.8±1.4	1.9±1.4	0.03	1.8±1.2	1.9±1.2	0.15	1.8±1.5	1.9±1.6	0.12
**Education, %**										
None/<br>NVQ/HND/HNC/CSEs<br>O-Level/ GCSEs<br>A-Levels/AS-Levels<br>College/University/ Professional	36.4<br>19.2<br>9.9<br>34.5	34.4<br>19.8<br>9.9<br>35.9	41.3<br>17.7<br>9.8<br>31.2	<0.001*	31.2<br>20.8<br>10.5<br>37.5	37.5<br>19.2<br>10.6<br>32.6	0.02	38<br>18.6<br>9.2<br>34.2	44.8<br>16.3<br>9.1<br>29.8	0.02
**Deprivation indices:**										
Education Score										
	18.6±17.9	17.7±17.4	20.6±19.3	<0.001*	17.6±17.1	20.9±19.3	<0.001*	17.9±17.6	20.4±18.9	0.003
Environmental Score										
	20.6±16.4	20±15.7	22.1±17.8	<0.001*	19.9±15.6	21.3±17.9	0.08	20.1±15.9	22.7±17.6	0.001*
Employment Score										
	0.1±0.06	0.09±0.06	0.1±0.07	<0.001*	0.09±0.06	0.1±0.07	<0.001*	0.10±0.06	0.11±0.07	0.01
Health Score										
	0.09±0.9	0.05±0.9	0.2±0.8	<0.001*	0.05±0.8	0.1±0.9	0.02	0.06±0.9	0.2±0.08	<0.001*

For peer review only - http://bmjopen.bmj.com/site/about/guidelines.xhtml

Housing Score										
	19.7±10.2	19.8±10.3	19.7±9.9	0.7	19.8±10.2	19.9±9.9	0.7	19.8±10.5	19.4±9.9	0.4
Income Score										
	0.1±0.11	0.1±0.1	0.1±0.11	<0.001*	0.1±0.1	0.1±0.12	<0.001*	0.1±0.11	0.1±0.11	0.008
**Income, %**										
1= <18K	32	31.5	33.5	0.1	31.2	32.1	0.17	31.8	34.7	0.26
2=18-30.9K	24.6	24.5	24.6		23.3	24		25.8	25.2	
3=31-51.9K	22.3	22.2	22.6		23.2	26.5		21.1	19.1	
4=52-100K	16.5	16.7	16		17.5	14.3		16	17.5	
5=>100K	4.6	5.1	3.3		4.8	3.2		5.3	3.5	
**Number in Household**										
	2.4±1.5	2.3±1.5	2.5±1.3	0.001*	2.4±1.8	2.5±1.4	0.04	2.3±1.1	2.4±1.3	0.006
**House Ownership, %**										
	81.4	82.2	79.6	0.04	84.5	80.4	0.02	79.6	78.8	0.7
**Neuroticism Score**										
	4.3±3.3	4.3±3.3	4.2±3.3	0.4	4.8±3.3	4.8±3.2	0.9	3.8±3.2	1.7±3.3	0.5
**Risk Taking Behavior, %**										
	30.7	29.9	32.5	0.1	23.3	27	0.7	37.3	37.5	0.9
**Leisure/Social Activities, %**										
0=No	32.4	32.7	31.8	0.003	32.9	34.3	0.03	32.4	29.6	0.09
1=Sports Club or Gym	24.1	24.6	23		25.8	21.9		23.2	24.1	
2=Pub or Social Club	21.1	20.4	22.6		13.6	16.1		28	28.6	
3=Religious Group	10.4	9.5	12.5		11.5	14.8		7.3	10.4	
4=Adult Education Class	2.8	3.1	2.2		4.4	3.7		1.5	0.9	
5= Other Group Activity	9.1	9.7	7.8		11.8	9.3		7.5	6.4	

Results are presented as mean ± standard deviation for numerical variables and percentage for categorical variables.

****Bonferroni Correction=0.05/18 (number of variables) =0.003, Based on this correction the differences marked with * are considered statistically significant***

For peer review only - http://bmjopen.bmj.com/site/about/guidelines.xhtml

Table 3. Summary of GBM model performance for predicting test-positive results in training (10-fold cross-validation) and test sets

		Accuracy	AUC	Threshold	PRAUC	Precision	Recall	Specificity
**Overall**	Test Set	0.56	0.570 (0.537-0.604)	0.25	0.33	0.34	0.56	0.56
**Male**	Test Set	0.53	0.575 (0.529-0.621)	0.32	0.38	0.37	0.67	0.46
**Female**	Test Set	0.62	0.561 (0.512-0.609)	0.26	0.31	0.32	0.35	0.72

**Area Under Curve (AUC) is reported with 95% Confidence Interval (CI)*
*** Used the best threshold of ROC as cut off for determining precision/recall, sensitivity/specificity*

Table4. Summary of GBM model performance for predicting being hospitalized in test-positive patients in training (10-fold cross-validation) and test sets

		Accuracy	AUC	Threshold	PRAUC	Precision	Recall	Specificity
**Overall**	Test Set	0.61	0.60 (0.534-0.665)	0.69	0.75	0.77	0.66	0.48
**Male**	Test Set	0.51	0.544 (0.453-0.635)	0.75	0.76	0.85	0.43	0.75
**Female**	Test Set	0.55	0.612 (0.532-0.692)	0.68	0.75	0.81	0.47	0.75

**Area Under Curve (AUC) is reported with 95% Confidence Interval (CI)*
*** Used the best threshold of ROC as cut off for determining precision/recall, sensitivity/specificity*

For peer review only - http://bmjopen.bmj.com/site/about/guidelines.xhtml

Figure Legends:

Figure 1: Overall Results: Partial dependency plots for predicting test positive results for first 10 most influential variables using permutation methods in the overall population using Shrinkage=0.01, bag fraction=0.5, interaction depth=5, CV fold=10, train fraction=0.7, n.minobsinnode = 10 as hyperparameters from grid search.

*The X axis is the predictor variable in the model. The Y axis shows how the predicted value changes with change in predictor variables. If the line in the plot is constant near zero it means that the variable has no effect on the model. A negative value means that a specific value of the predictor variable is less likely to predict the correct class of outcome, whereas a positive value means the predictor variable has a positive impact on predicting the correct class. A LOESS (locally estimated scatterplot smoothing) line is fit to show the trend.

** The number beside each variable shows the order of feature importance and most influential variables for each model. For identifying the most influential variables, permutation-based feature importance was utilized. This approach measures a feature (variable) importance by calculating the increase of the model's prediction error after permuting the feature.

Figure 2: Results for Females: Partial dependency plots for predicting test positive results for first 5 most influential variables using permutation methods in Females using Shrinkage=0.01, bag fraction=1, interaction depth=7, CV fold=10, train fraction=0.8, n.minobsinnode = 15 as hyperparameters from grid search.

Figure 3: Results for Males: Partial dependency plots for predicting test positive results for first 5 most influential variables using permutation methods in males using, Shrinkage=0.001, bag fraction=1, interaction depth=3, CV fold=10, train fraction=0.8, n.minobsinnode = 5 as hyperparameters from grid search

Figure 4: Hospitalization in overall population: Partial dependency plots for predicting hospitalization in test positive patients for first 5 most influential variables using permutation methods using, Shrinkage=0.1, bag fraction=0.8, interaction depth=3, CV fold=10, train fraction=0.7, n.minobsinnode = 15 as hyperparameters from grid search

Figure 5: Hospitalization of females: Partial dependency plot for predicting hospitalization in test positive females for first 5 most influential variables using permutation methods using, Shrinkage=0.05, bag fraction=0.65, interaction depth=7, CV fold=10, train fraction=0.8, n.minobsinnode = 15 as hyperparameters from grid search

Figure 6: Hospitalization of males: Partial dependency plot for predicting hospitalized patients in test positive males for first 5 most influential variables using permutation methods using, Shrinkage=0.1, bag fraction=0.5, interaction depth=5, CV fold=10, train fraction=0.8, n.minobsinnode = 15 as hyperparameters from grid search

For peer review only - http://bmjopen.bmj.com/site/about/guidelines.xhtml

References:

1. Johns Hopkins University J. COVID-19 Dashboard by the Center for Systems Science and Engineering (CSSE) 2021 [updated 11/21/2021. Available from: https://www.arcgis.com/apps/opsdashboard/index.html#/bda7594740fd40299423467b48e9ecf6.
2. Niedzwiedz CL, O'Donnell CA, Jani BD, et al. Ethnic and socioeconomic differences in SARS-CoV-2 infection: prospective cohort study using UK Biobank. BMC Medicine 2020;**18**(1):1-14.
3. Lassale C, Gaye B, Hamer M, et al. Ethnic Disparities in Hospitalization for COVID-19: a Community-Based Cohort Study in the UK. Brain Behav Immun 2020;**88**:44-49.
4. Prats-Uribe A, Paredes R, Prieto-Alhambra D. Ethnicity, comorbidity, socioeconomic status, and their associations with COVID-19 infection in England: a cohort analysis of UK Biobank data. medRxiv 2020.
5. Kolin DA, Kulm S, Elemento O. Clinical and Genetic Characteristics of Covid-19 Patients from UK Biobank. Plos one 2020;**15**(11):e0241264.
6. Hamer M, Kivimäki M, Gale CR, et al. Lifestyle Risk Factors, Inflammatory Mechanisms, and COVID-19 Hospitalization: A Community-Based Cohort Study of 387,109 Adults in UK. Brain, Behavior, and Immunity 2020;**87**:184-7.
7. Patel AP, Paranjpe MD, Kathiresan NP, et al. Race, Socioeconomic Deprivation, and Hospitalization for COVID-19 in English participants of a National Biobank. International journal for equity in health 2020;**19**(1):1-4.
8. Darling AL, Ahmadi KR, Ward KA, et al. Vitamin D status, body mass index, ethnicity and COVID-19: Initial analysis of the first-reported UK Biobank COVID-19 positive cases (n 580) compared with negative controls (n 723). Proceedings of the Nutrition Society 2020;**80**(OCE1):E17.
9. Schiffer VM, Janssen EB, van Bussel BC, et al. The" sex gap" in COVID-19 trials: a scoping review. EClinicalMedicine 2020;**29-30**:100652.
10. Tadiri CP, Gisinger T, Kautzy-Willer A, et al. The influence of sex and gender domains on COVID-19 cases and mortality. CMAJ 2020;**192**(36):E1041-E45.
11. Scully EP, Haverfield J, Ursin RL, et al. Considering how biological sex impacts immune responses and COVID-19 outcomes. Nature Reviews Immunology 2020;**20**:442–47.
12. Galbadage T, Peterson BM, Awada J, et al. Systematic Review and Meta-Analysis of Sex-Specific COVID-19 Clinical Outcomes. Front Med (Lausanne) 2020;**23**(7):348.
13. Haitao T, Vermunt JV, Abeykoon J, et al. COVID-19 and Sex Differences: Mechanisms and Biomarkers. Mayo Clinic proceedings 2020;**95**(10):2189-203.
14. Johnson JL, Greaves L, Repta R. Better science with sex and gender: Facilitating the use of a sex and gender-based analysis in health research. International Journal for Equity in Health 2009;**8**(1):1-11.
15. CIHR OTM. Online Training Modules: Integrating Sex & Gender in Health Research 2021 [updated 12/07/2020. Available from: https://cihr-irsc.gc.ca/e/49347.html.
16. World Health Organization (WHO) S. Addressing sex and gender in epidemic-prone infectious diseases 2007 [updated 12/07/2020. Available from: https://www.who.int/csr/resources/publications/SexGenderInfectDis.pdf.
17. Al Youha S, Alkhamis M, Al Mazeedi S, et al. Using Machine Learning to Unveil Demographic and Clinical Features of COVID-19 Symptomatic and Asymptomatic Patients. 2020.
18. Wong KC, Xiang Y, Yin L, et al. Uncovering clinical risk factors and prediction of severe COVID-19: A machine learning approach based on UK Biobank data. JMIR Public Health Surveill 2021;**7**(8):e29544.
19. Ahamad MM, Aktar S, Rashed-Al-Mahfuz M, et al. A machine learning model to identify early stage symptoms of SARS-Cov-2 infected patients. Expert systems with applications 2020;**160**:113661.
20. Sudlow C, Gallacher J, Allen N, et al. UK biobank: an open access resource for identifying the causes of a wide range of complex diseases of middle and old age. Plos med 2015;**12**(3):e1001779.
21. Armstrong J, Rudkin JK, Allen N, et al. Dynamic linkage of covid-19 test results between public health england's second generation surveillance system and uk biobank. Microbial genomics 2020;**6**(7).
22. Mutambudzi M, Niedzwiedz CL, Macdonald EB, et al. Occupation and risk of COVID-19: prospective cohort study of 120,621 UK Biobank participants. Occup Environ Med 2020;**78**(5):307–14.

For peer review only - http://bmjopen.bmj.com/site/about/guidelines.xhtml

23. Pilote L, Norris CM, Raparelli V, et al. Gender Outcomes INternational Group: to Further Well-being Development (GOING-FWD): https://www.mcgill.ca/going-fwd4gender/, 2020.
24. Tadiri CP, Raparelli V, Abrahamowicz M, et al. Methods for prospectively incorporating gender into health sciences research. J Clin Epidemiol 2021;**129**:191-97.
25. Cho ER, Slutsky AS, Jha P. Smoking and the risk of COVID-19 infection in the UK Biobank Prospective Study. medRxiv 2020.
26. Hastie CE, Mackay DF, Ho F, et al. Vitamin D concentrations and COVID-19 infection in UK Biobank. Diabetes Metab Syndr 2020;**14**(5):1315-16.
27. Khawaja AP, Warwick AN, Hysi PG, et al. Associations with covid-19 hospitalisation amongst 406,793 adults: the UK Biobank prospective cohort study. medRxiv 2020.
28. Friedman JH. Greedy function approximation: a gradient boosting machine. Annals of statistics 2001:1189-232.
29. Ridgeway G. Generalized Boosted Models: A guide to the gbm package. Update 2007;**1**(1):2007.
30. Bühlmann P, Hothorn T. Boosting algorithms: Regularization, prediction and model fitting. Statistical Science 2007;**22**(4):477-505.
31. Maleki F, Ovens K, Najafian K, et al. Overview of Machine Learning Part 1: Fundamentals and Classic Approaches. Neuroimaging Clin N Am 2020;**30**(4):e17-e32.
32. Maleki F, Muthukrishnan N, Ovens K, et al. Machine Learning Algorithm Validation: From Essentials to Advanced Applications and Implications for Regulatory Certification and Deployment. Neuroimaging Clin N Am 2020;**30**(4):433-45.
33. Yan Y. MLmetrics: Machine learning evaluation metrics. R package version 2016;**1**(1).
34. Davis J, Goadrich M. The relationship between Precision-Recall and ROC curves. Proceedings of the 23rd international conference on Machine learning. Pittsburgh, Pennsylvania, USA: Association for Computing Machinery, 2006:233–40.
35. Boyd K, Costa VS, Davis J, et al. Unachievable region in precision-recall space and its effect on empirical evaluation. Proc Int Conf Mach Learn 2012;**2012**:349.
36. Nicodemus KK, Malley JD, Strobl C, et al. The behaviour of random forest permutation-based variable importance measures under predictor correlation. BMC bioinformatics 2010;**11**(1):1-13.
37. Greenwell BM. pdp: An R Package for Constructing Partial Dependence Plots. R J 2017;**9**(1):421.
38. Khoda Bakhshi A, Ahmed MM. Utilizing black-box visualization tools to interpret non-parametric real-time risk assessment models. Transportmetrica A: Transport Science 2021;**17**(4):739-65.
39. Gebhard C, Regitz-Zagrosek V, Neuhauser HK, et al. Impact of sex and gender on COVID-19 outcomes in Europe. Biology of Sex Differences 2020;**11**(1):1-13.
40. Yates T, Razieh C, Zaccardi F, et al. Obesity and risk of COVID-19: analysis of UK Biobank. Primary Care Diabetes 2020;**14**:566-67.
41. Atkins JL, Masoli JA, Delgado J, et al. Preexisting comorbidities predicting severe COVID-19 in older adults in the UK Biobank community cohort. J Gerontol A Biol Sci Med Sci 2020;**75**(11):2224-30.
42. Guo W, Li M, Dong Y, et al. Diabetes is a risk factor for the progression and prognosis of COVID-19. Diabetes/metabolism research and reviews 2020;**36**(7):e3319.
43. Scalsky RJ, Desai K, Chen Y-J, et al. Baseline Cardiometabolic Profiles and SARS-CoV-2 Risk in the UK Biobank. PLoS ONE 2020;**16**(4):e0248602.
44. Aldridge RW, Lewer D, Katikireddi SV, et al. Black, Asian and Minority Ethnic groups in England are at increased risk of death from COVID-19: indirect standardisation of NHS mortality data. Wellcome Open Research 2020;**5**(88):88.

For peer review only

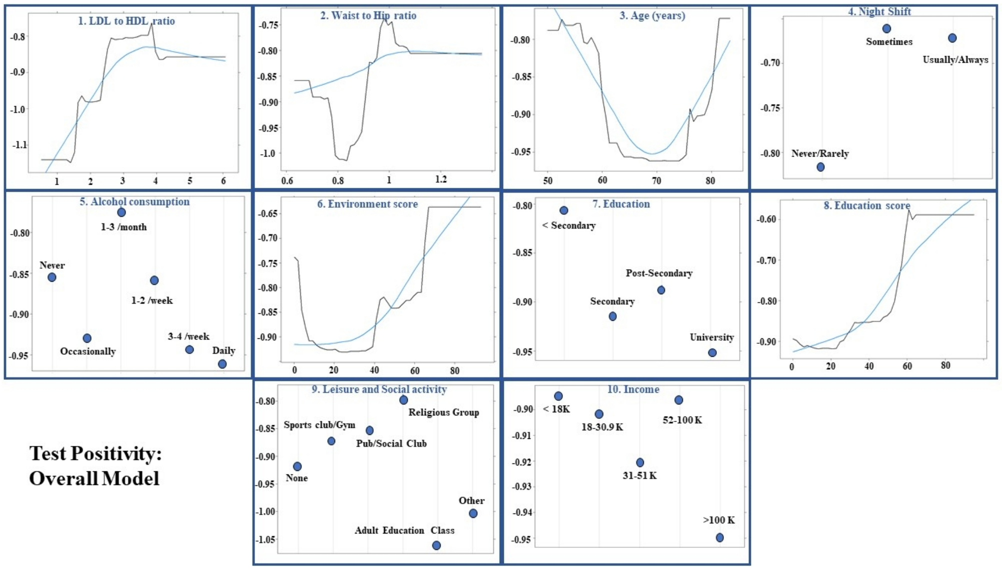

Figure 1: Overall Results: Partial dependency plots for predicting test positive results for first 10 most influential variables using permutation methods in the overall population using Shrinkage=0.01, bag fraction=0.5, interaction depth=5, CV fold=10, train fraction=0.7, n.minobsinnode = 10 as hyperparameters from grid search.

*The X axis is the predictor variable in the model. The Y axis shows how the predicted value changes with change in predictor variables. If the line in the plot is constant near zero it means that the variable has no effect on the model. A negative value means that a specific value of the predictor variable is less likely to predict the correct class of outcome, whereas a positive value means the predictor variable has a positive impact on predicting the correct class. A LOESS (locally estimated scatterplot smoothing) line is fit to show the trend.

** The number beside each variable shows the order of feature importance and most influential variables for each model. For identifying the most influential variables, permutation-based feature importance was utilized. This approach measures a feature (variable) importance by calculating the increase of the model's prediction error after permuting the feature.

338x190mm (96 x 96 DPI)

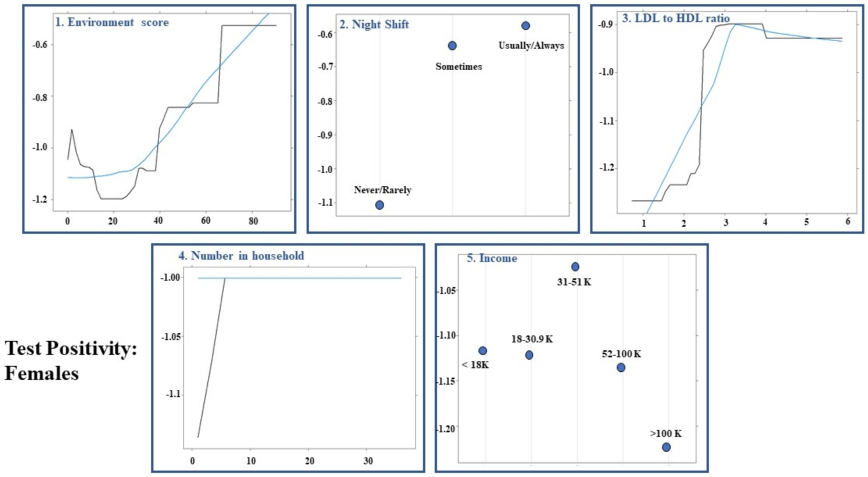

Figure 2: Results for Females: Partial dependency plots for predicting test positive results for first 5 most influential variables using permutation methods in Females using Shrinkage=0.01, bag fraction=1, interaction depth=7, CV fold=10, train fraction=0.8, n.minobsinnode = 15 as hyperparameters from grid search.

338x190mm (96 x 96 DPI)

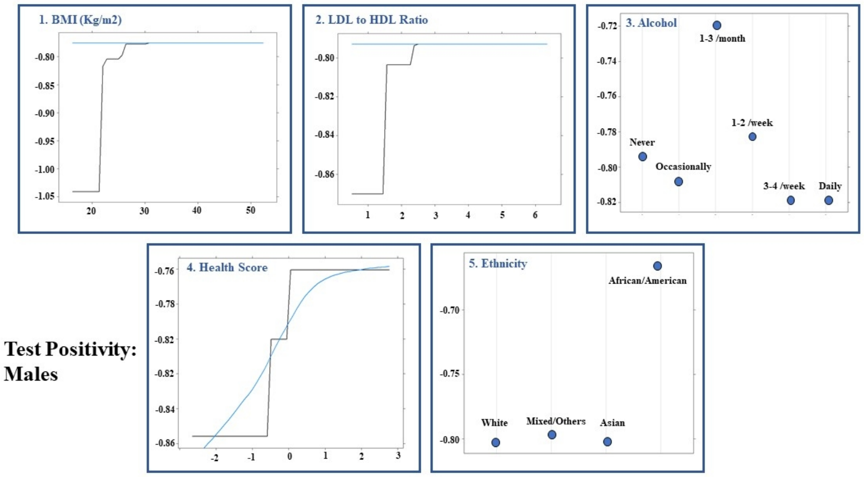

Figure 3: Results for Males: Partial dependency plots for predicting test positive results for first 5 most influential variables using permutation methods in males using, Shrinkage=0.001, bag fraction=1, interaction depth=3, CV fold=10, train fraction=0.8, n.minobsinnode = 5 as hyperparameters from grid search

338x190mm (96 x 96 DPI)

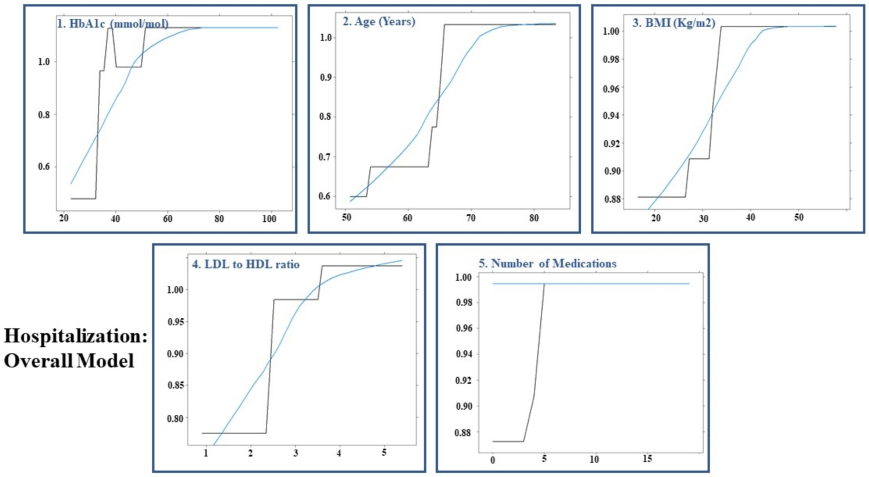

Figure 4: Hospitalization in overall population: Partial dependency plots for predicting hospitalization in test positive patients for first 5 most influential variables using permutation methods using, Shrinkage=0.1, bag fraction=0.8, interaction depth=3, CV fold=10, train fraction=0.7, n.minobsinnode = 15 as hyperparameters from grid search

338x190mm (96 x 96 DPI)

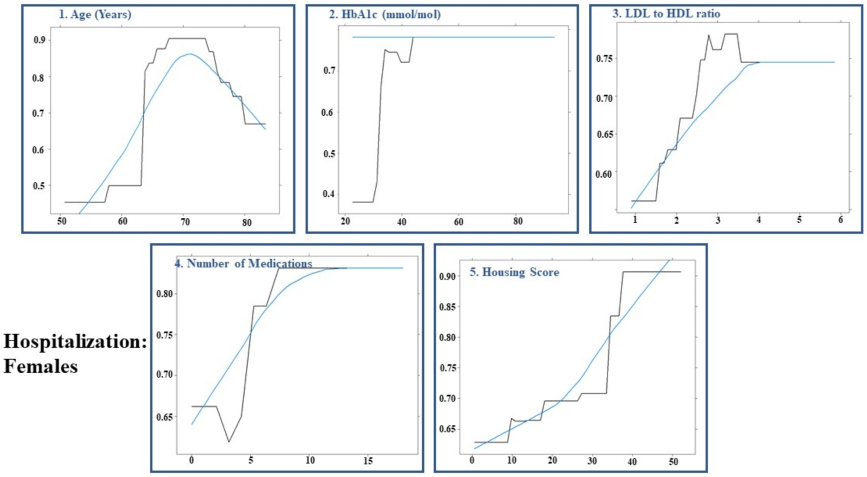

Figure 5: Hospitalization of females: Partial dependency plot for predicting hospitalization in test positive females for first 5 most influential variables using permutation methods using, Shrinkage=0.05, bag fraction=0.65, interaction depth=7, CV fold=10, train fraction=0.8, n.minobsinnode = 15 as hyperparameters from grid search

338x190mm (96 x 96 DPI)

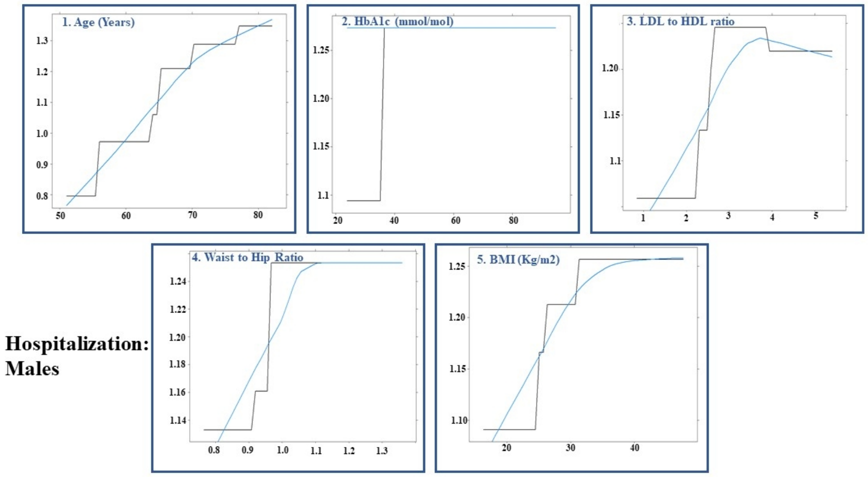

Figure 6: Hospitalization of males: Partial dependency plot for predicting hospitalized patients in test positive males for first 5 most influential variables using permutation methods using, Shrinkage=0.1, bag fraction=0.5, interaction depth=5, CV fold=10, train fraction=0.8, n.minobsinnode = 15 as hyperparameters from grid search

338x190mm (96 x 96 DPI)

Supplemental

Supplemental 1. Definition of deprivation indices

Variable Name	**Definition**
Education score	This measure shows the extent of deprivation in education, skills and training in an area within 2 sub domains including children and young people and adults skills.
Living environment score	This score measures the quality of individuals' immediate surroundings both within and outside the home within the 'indoors' living environment, which measures the quality of housing, and the 'outdoors' living environment which contains two measures relating to air quality and road traffic accidents subdomains.
Employment score	This score measures employment deprivation in an area conceptualized as involuntary exclusion of the working age population from the labour market.
Income score	This score measures the proportion of the population in an area experiencing deprivation related to low income.
Housing score	This score measures the physical and financial accessibility of housing and key local services. The indicators fall into two sub-domains: 'geographical barriers', which relate to the physical proximity of local services, and 'wider barriers' which includes issues relating to access to housing such as affordability.
Health score	This score measures premature death and the impairment of quality of life by poor health. It considers both physical and mental health. The domain measures morbidity, disability and premature mortality but not aspects of behaviour or environment that may be predictive of future health deprivation.

For peer review only - http://bmjopen.bmj.com/site/about/guidelines.xhtml

STROBE 2007 (v4) checklist of items to be included in reports of observational studies in epidemiology*

Checklist for cohort, case-control, and cross-sectional studies (combined)

Section/Topic	Item #	Recommendation	Reported on page #
**Title and abstract**	1	(*a*) Indicate the study's design with a commonly used term in the title or the abstract	1-2
		(*b*) Provide in the abstract an informative and balanced summary of what was done and what was found	1-2
**Introduction**			
Background/rationale	2	Explain the scientific background and rationale for the investigation being reported	4-5
Objectives	3	State specific objectives, including any pre-specified hypotheses	4-5
**Methods**			
Study design	4	Present key elements of study design early in the paper	5
Setting	5	Describe the setting, locations, and relevant dates, including periods of recruitment, exposure, follow-up, and data collection	5
Participants	6	(*a*) *Cohort study*—Give the eligibility criteria, and the sources and methods of selection of participants. Describe methods of follow-up<br>*Case-control study*—Give the eligibility criteria, and the sources and methods of case ascertainment and control selection. Give the rationale for the choice of cases and controls<br>*Cross-sectional study*—Give the eligibility criteria, and the sources and methods of selection of participants	5
		(*b*) *Cohort study*—For matched studies, give matching criteria and number of exposed and unexposed<br>*Case-control study*—For matched studies, give matching criteria and the number of controls per case	
Variables	7	Clearly define all outcomes, exposures, predictors, potential confounders, and effect modifiers. Give diagnostic criteria, if applicable	6-7
Data sources/ measurement	8*	For each variable of interest, give sources of data and details of methods of assessment (measurement). Describe comparability of assessment methods if there is more than one group	*6-7*
Bias	9	Describe any efforts to address potential sources of bias	NA
Study size	10	Explain how the study size was arrived at	5
Quantitative variables	11	Explain how quantitative variables were handled in the analyses. If applicable, describe which groupings were chosen and why	7
Statistical methods	12	(*a*) Describe all statistical methods, including those used to control for confounding	7-8
		(*b*) Describe any methods used to examine subgroups and interactions	7-8
		(*c*) Explain how missing data were addressed	7
		(*d*) *Cohort study*—If applicable, explain how loss to follow-up was addressed<br>*Case-control study*—If applicable, explain how matching of cases and controls was addressed	

For peer review only

		Cross-sectional study—If applicable, describe analytical methods taking account of sampling strategy	
		(*e*) Describe any sensitivity analyses	7-8
**Results**			
Participants	13*	(a) Report numbers of individuals at each stage of study—eg numbers potentially eligible, examined for eligibility, confirmed eligible, included in the study, completing follow-up, and analysed	9
		(b) Give reasons for non-participation at each stage	NA
		(c) Consider use of a flow diagram	NA
Descriptive data	14*	(a) Give characteristics of study participants (eg demographic, clinical, social) and information on exposures and potential confounders	9
		(b) Indicate number of participants with missing data for each variable of interest	-
		(c) *Cohort study*—Summarise follow-up time (eg, average and total amount)	-
Outcome data	15*	*Cohort study*—Report numbers of outcome events or summary measures over time	-
		Case-control study—Report numbers in each exposure category, or summary measures of exposure	-
		Cross-sectional study—Report numbers of outcome events or summary measures	9-10
Main results	16	(*a*) Give unadjusted estimates and, if applicable, confounder-adjusted estimates and their precision (eg, 95% confidence interval). Make clear which confounders were adjusted for and why they were included	9-10
		(*b*) Report category boundaries when continuous variables were categorized	9-10
		(*c*) If relevant, consider translating estimates of relative risk into absolute risk for a meaningful time period	9-10
Other analyses	17	Report other analyses done—eg analyses of subgroups and interactions, and sensitivity analyses	9-10
**Discussion**			
Key results	18	Summarise key results with reference to study objectives	10-11
Limitations	19	Discuss limitations of the study, taking into account sources of potential bias or imprecision. Discuss both direction and magnitude of any potential bias	13
Interpretation	20	Give a cautious overall interpretation of results considering objectives, limitations, multiplicity of analyses, results from similar studies, and other relevant evidence	14
Generalisability	21	Discuss the generalisability (external validity) of the study results	14
**Other information**			
Funding	22	Give the source of funding and the role of the funders for the present study and, if applicable, for the original study on which the present article is based	14

*Give information separately for cases and controls in case-control studies and, if applicable, for exposed and unexposed groups in cohort and cross-sectional studies.

Note: An Explanation and Elaboration article discusses each checklist item and gives methodological background and published examples of transparent reporting. The STROBE checklist is best used in conjunction with this article (freely available on the Web sites of PLoS Medicine at http://www.plosmedicine.org/, Annals of Internal Medicine at http://www.annals.org/, and Epidemiology at http://www.epidem.com/). Information on the STROBE Initiative is available at www.strobe-statement.org.

For peer review only

BMJ Open

The Importance of Sex and Gender Factors for COVID-19 Infection and Hospitalization: A Sex-stratified Analysis Using Machine Learning in UK Biobank data

Journal:	*BMJ Open*
Manuscript ID	bmjopen-2021-050450.R2
Article Type:	Original research
Date Submitted by the Author:	02-Mar-2022
Complete List of Authors:	Azizi, Zahra; McGill University Health Centre<br>Shiba, Yumika; McGill University, Department of Biology<br>Alipour, Pouria; McGill University Health Centre; McGill University<br>Maleki, Farhad; McGill University Health Centre, Department of Radiology<br>Raparelli, Valeria; University of Ferrara, Department of Translational Medicine; University of Alberta, Department of Translational Medicine<br>Norris, Colleen; University of Alberta, Faculty of Nursing<br>Forghani, Reza; McGill University Health Centre, Department of Radiology<br>Pilote, Louise; McGill University Health Centre; McGill University<br>El Emam, Khaled; University of Ottawa; University of Ottawa<br>Investigators, GOING FWD ; McGill University
**Primary Subject Heading**:	Public health
Secondary Subject Heading:	Infectious diseases
Keywords:	Health policy < HEALTH SERVICES ADMINISTRATION & MANAGEMENT, Risk management < HEALTH SERVICES ADMINISTRATION & MANAGEMENT, COVID-19

SCHOLARONE™
Manuscripts

I, the Submitting Author has the right to grant and does grant on behalf of all authors of the Work (as defined in the below author licence), an exclusive licence and/or a non-exclusive licence for contributions from authors who are: i) UK Crown employees; ii) where BMJ has agreed a CC-BY licence shall apply, and/or iii) in accordance with the terms applicable for US Federal Government officers or employees acting as part of their official duties; on a worldwide, perpetual, irrevocable, royalty-free basis to BMJ Publishing Group Ltd ("BMJ") its licensees and where the relevant Journal is co-owned by BMJ to the co-owners of the Journal, to publish the Work in this journal and any other BMJ products and to exploit all rights, as set out in our licence.

The Submitting Author accepts and understands that any supply made under these terms is made by BMJ to the Submitting Author unless you are acting as an employee on behalf of your employer or a postgraduate student of an affiliated institution which is paying any applicable article publishing charge ("APC") for Open Access articles. Where the Submitting Author wishes to make the Work available on an Open Access basis (and intends to pay the relevant APC), the terms of reuse of such Open Access shall be governed by a Creative Commons licence – details of these licences and which Creative Commons licence will apply to this Work are set out in our licence referred to above.

Other than as permitted in any relevant BMJ Author's Self Archiving Policies, I confirm this Work has not been accepted for publication elsewhere, is not being considered for publication elsewhere and does not duplicate material already published. I confirm all authors consent to publication of this Work and authorise the granting of this licence.

For peer review only

The Importance of Sex and Gender Factors for COVID-19 Infection and Hospitalization: A Sex-stratified Analysis Using Machine Learning in UK Biobank data

Zahra Azizi, MD, MSc [1]; Yumika Shiba [2]; Pouria Alipour, MSc[1,3]; Farhad Maleki, PhD[4]; Valeria Raparelli, MD, PhD [5,6]; Colleen Norris, PhD [6,7]; Reza Forghani, MD, PhD[4]; Louise Pilote, MD, PhD* [1,3,8]; Khaled El-Emam, PhD* [9,10,11] and the GOING FWD Investigators

***Drs Pilote and El-Emam are co-senior authors**

1. Centre for Outcomes Research and Evaluation, McGill University Health Centre Research Institute, Montreal, QC, Canada
2. Department of Biology; McGill University, Montreal, QC, Canada
3. Faculty of Medicine and Health Sciences, McGill University, Montreal, QC, Canada
4. Augmented Intelligence & Precision Health Laboratory (AIPHL), Department of Radiology, McGill University Health Centre, Montreal, QC, Canada
5. Department of Translational Medicine, University of Ferrara, Ferrara, Italy
6. Faculty of Nursing, University of Alberta, Edmonton, Alberta, Canada
7. Heart and Stroke Strategic Clinical Networks-Alberta Health Services, Alberta, Canada
8. Divisions of Clinical Epidemiology and General Internal Medicine, McGill University Health Centre Research Institute, Montreal, QC, Canada
9. Children's Hospital of Eastern Ontario Research Institute, Ottawa, Ontario, Canada
10. Faculty of Medicine, University of Ottawa, Ottawa, Ontario. Canada
11. Replica Analytics Ltd, Ottawa, Ontario, Canada

Corresponding Authors:

Louise Pilote MD, MPH, PhD
Center for Outcomes Research and Evaluation
Divisions of Clinical Epidemiology and General Internal Medicine
McGill University Health Centre Research Institute
5252 boulevard de Maisonneuve, Montreal
Quebec, H3A 1A1, Canada
Tel: 514 934-1934 ext. 44722; Fax: 514 843-1676
E-mail: louise.pilote@mcgill.ca

Khaled El Emam, PhD
Children's Hospital of Eastern Ontario Research Institute
401 Smyth Road, Ottawa
Ontario K1J 8L1, Canada
E-mail: kelemam@ehealthinformation.ca
Tel: +1 613-797-5412

Word Count: 3781 (w/o abstract and references)

For peer review only - http://bmjopen.bmj.com/site/about/guidelines.xhtml

Abstract:

Objective: To examine sex and gender roles in COVID-19 test positivity and hospitalization in sex-stratified predictive models using machine learning.

Design: Cross-sectional study

Setting: UK Biobank prospective cohort

Participants: Participants tested between March 16th, 2020, to May 18th, 2020, were analyzed.

Main outcome measures: The endpoints of the study were COVID-19 test positivity and hospitalization. Forty-two individuals' demographics, psychosocial factors, and comorbidities were used as likely determinants of outcomes. Gradient boosting machine was utilized for building prediction models.

Results: Of 4,510 individuals tested (51.2% females, mean age= 68.5±8.9 years), 29.4% tested positive. Males were more likely to be positive than females (31.6% vs 27.3%, P=0.001). In females, living in more deprived areas, lower income, increased LDL-to-HDL ratio, working night shifts, and living with a greater number of family members were associated with a higher likelihood of COVID-19 positive test. While in males, greater body mass index and LDL-to-HDL ratio were the factors associated with a positive test. Older age, and adverse cardio-metabolic characteristics were the most prominent variables associated with hospitalization of test positive patients in both overall, and sex-stratified models.

Conclusion: High-risk jobs, crowded living arrangements, and living in deprived areas were associated with increased COVID-19 infection in females, while high-risk cardio-metabolic characteristics were more influential in males. Gender-related factors have a greater impact in females; hence they should be considered in identifying priority groups for COVID-19 infection vaccination campaigns.

Keywords: Covid-19 infection, SARS-Cov-2, Test Positive, Hospitalization, Sex, Gender, Machine

For peer review only - http://bmjopen.bmj.com/site/about/guidelines.xhtml

Learning, Vaccination

Strengths and limitations:

- A unique feature of the study is the investigation of numerous psycho-socio-cultural factors in conjunction with clinical and laboratory factors made feasible through machine learning algorithms.
- The assessment of sex-stratified algorithms to elucidate the most influential factors in both sexes adds novel information as it has rarely been done to date.
- The first limitation of this study is selection bias due to the lack of systematic and random testing across the UK.
- This analysis was done using the baseline diagnostic data that was collected from 2006 to 2010; therefore, misclassification of determinants is possible. However, previous studies of the UK Biobank have shown a high correlation between baseline and follow-up data for a subsample of patients who had further visits for imaging.
- Finally, the relatively low predictive model performance (Test positivity: AUC: 0.570 (95% Confidence Interval (CI): 0.537-0.604), hospitalization: AUC: 0.60 (95% CI: 0.534-0.665)) —as expected—reflects other influences not captured in the UK Biobank.

For peer review only

For peer review only - http://bmjopen.bmj.com/site/about/guidelines.xhtml

Introduction

The novel coronavirus SARS-CoV-2 (COVID-19) pandemic has led to more than 250 million reported positive cases and over five million deaths worldwide as of November 2021[1]. As vaccination is rolling out, continuous efforts are made to establish risk factors for the disease and find vulnerable populations across the globe [2-8].

There has been an imbalance in infection susceptibility, severity, and mortality between males and females [9]. These differences are multifactorial and have been attributed to a combination of biological (i.e., genetic, hormonal) and psycho-socio-cultural differences (gender) [9-13]. While "sex" refers to a set of biological attributes in humans and animals, "gender" refers to the roles, behaviours, and identities of individuals that forms throughout life [14 15]. It is increasingly recognized that both sex and gender play significant roles in health outcomes [14 15], including the acquisition of infections and response to infection and treatments [16]. The World Health Organization (WHO) statement [16], addressing sex and gender in epidemic-prone infectious diseases, outlines that differences between males and females can lead to differences in activity patterns in work and in family roles, which may increase the risk of exposure to infectious disease in a particular setting [16]. Therefore, lack of consideration of the influences of sex and gender in COVID-19 contraction can potentially hinder the effectiveness of COVID-19 vaccination prioritization strategies.

With the introduction of the COVID-19 vaccines, identifying those most vulnerable to infection with accurate prediction models that account for more complex relationships is urgent. Machine learning algorithms can explore non-linear and complex relationships by considering the interaction between both biological and psycho-socio-cultural factors together; however, these methods are still underutilized in the medical field, and few predictive models have been tailored

For peer review only - http://bmjopen.bmj.com/site/about/guidelines.xhtml

to each sex [17-19]. Therefore, we examined sex and gender-related factors associated with SARS-CoV-2 test positivity and COVID-19 hospitalization in the UK Biobank cohort and developed sex-stratified predictive models using machine learning methods.

Methods

Study Population

This is a cross sectional study of UK Biobank data. The UK Biobank (UKB, https://www.ukbiobank.ac.uk/) is a prospective cohort study that collects health, lifestyle, genetic, and imaging data for over 500,000 randomly selected participants in the UK [20]. Baseline data collection took place between 2006–2010 across England, Scotland, and Wales. The age of participants at recruitment was between 40 and 69 years [20]. Data was collected in 22 Assessment Centers through 4 main methods; touchscreen questionnaires, verbal interviews, physical measures, and biological sampling [20]. For this study, only data from England between March 16th, 2020, to May 18th, 2020, was used due to the unavailability of COVID-19 test results from Scotland and Wales at the time of analysis.

Ethics approval for the project was obtained from the coordinator center at McGill University, Canada (REB # 2020-5452 (Gender Outcomes INternational Group: to Further Well-being Development (GOING-FWD), CIHR GNP-161904, 2018/10/01 to 2023/03/31). The data was obtained from the UK Biobank repository (Application ID:45551).

Patient and Public Involvement

No patient involved in the design, or conduct, or reporting, or dissemination plans of our research.

COVID-19 Test Results

COVID-19 test results were available from Public Health England (PHE) data for 4510

For peer review only - http://bmjopen.bmj.com/site/about/guidelines.xhtml

participants , which was linked to UK Biobank baseline data [21]. The primary endpoint of the study was test positivity. We defined testing positive as having at least one positive test result. The test results were available from March 16th, 2020, to May 18th, 2020. The secondary endpoint of the study was being hospitalized for a COVID-19 related illness. For this purpose, we chose test-positive patients who had at least one positive result in an in-patient setting. The results are from samples taken from the combined nose, throat, sputum, or lower respiratory tract. The analysis for infection was done using real-time polymerase chain reaction (RT-PCR) tests for SARS-CoV-2.

Baseline Characteristics

Patients' demographics, psychosocial (gender-related variables), anthropometric variables, and comorbidities that were collected at baseline 2006-2010 were used for analysis. We used this particular baseline data for socioeconomic status and occupation since studies have shown a high correlation between baseline and follow-up data for a subsample of patients who had further visits for imaging [22].

Gender-related Psychosocial Variables

A multistep approach for identifying gender-related variables was exploited based on GOING-FWD (Gender Outcomes International Group to Further Well-being Development) methodology [23], the Women Health Research Network's gender framework (i.e. gender identity, gender roles, gender relationships, and institutionalized gender), and the Canadian Institutes of Health Research sex and gender modules [14 15 24]. Based on this approach and data availability, the following variables were selected: multiple deprivation indices (Supplemental 1), employment, jobs involving night shift, education level, smoking status, alcohol consumption, number of

For peer review only - http://bmjopen.bmj.com/site/about/guidelines.xhtml

children, household crowding, housing ownership, income, leisure and social activities, risk-taking behaviors, and neuroticism score. Details of the variables are available in the UK Biobank data dictionary (https://biobank.ctsu.ox.ac.uk/crystal/search.cgi).

Comorbidities

Baseline comorbidities were self-reported and collected using a touch screen device—i.e., hypertension (HTN), diabetes (DM), chronic obstructive pulmonary disease (COPD), asthma, allergy, history of stroke, heart attack, angina, deep vein thrombosis (DVT), pulmonary embolism (PE), and cancer (CA). In addition, the number of medications and long-standing illness, disability, or infirmity were also included and coded as dichotomous variables. These variables were selected based on their significance as demonstrated by a number of previous investigations on the UK Biobank [2 3 5-8 18 22 25-27].

Physical and Biological Characteristics

Variables used as measures of physical and biological characteristics were body mass index (BMI) (defined as weight (Kg)/height $(m)^2$), waist to hip ratio (WHR), levels of vitamin D, hemoglobin A1C (HbA1C), high-density lipoprotein (HDL) and low-density lipoprotein (LDL); all coded as continuous variables.

Statistical Analyses

Descriptive statistics were reported as mean and standard deviation for continuous variables and frequency and percentage for categorical variables. Group-based differences (test negative versus positive) in baseline characteristics were compared using independent Student's t-test for continuous variables (or its non-parametric counterpart for skewed distributions) and chi-square for categorical variables. P-values of less-than or equal-to 0.05 were considered statistically significant. A complete case analysis (pairwise deletion) approach was used for dealing with

For peer review only - http://bmjopen.bmj.com/site/about/guidelines.xhtml

missing data in the descriptive analysis. Bonferroni type adjustments were utilized to correct for multiple testing and the results after adjustment were used for interpretation. Data analysis was performed using R-Studio (Version 1.3.1093) and R software (v4.0.3).

Machine Learning

The data was split into 70% training and 30% test sets, where the test set was only used for the evaluation of the final models. The training set was used to develop gradient boosting decision tree models [28-30] using the "gbm3" R package for predicting SARS-Cov-2 test-positivity and hospitalization prediction. To reduce the effect of class imbalance on model development [31], a bootstrap oversampling approach was used. For each endpoint, i.e., test-positivity or hospitalization, three different models were developed: (1) a model for males, (2) a model for females, and (3) an overall model for males and females combined. Calibration was then performed using isotonic regression method.

Using k-fold cross-validation is considered a best practice in developing machine learning models [32]. Therefore, for developing each model, a 10-fold cross-validation was performed on the training set. We also used a grid-search procedure to find the best combination hyper-parameters (e.g., learning rate, interaction depth, bagging fraction, and the minimum number of observations in terminal nodes) (Supplement2) using **5-fold cross-validation on the training dataset** and the AUROC metric as the criterion. A Bernoulli distribution was used for classification models. These resulted in three trained models for SARS-Cov-2 test-positivity and three models for SARS-Cov-2 hospitalization.

To provide an unbiased estimate of the model generalization errors, the performance of the trained models was assessed and reported on the test set. Confusion matrix derived metrics including accuracy, precision, recall (sensitivity), specificity as well as AUC score were used as

For peer review only - http://bmjopen.bmj.com/site/about/guidelines.xhtml

the performance measures [33]. We used the best threshold of ROC as cut off for determining precision/recall, sensitivity/specificity. Another metric which focuses on predictions of the positive class is the Area Under the Precision-Recall Curve (AUPRC) [34]. Interpretation of AUPRC is dependent on the class distribution of the outcome as the minimal achievable value is dependent on that distribution [35], and the AUPRC value of a random classifier is the rate of the positive class [35].

Most Influential Variables

For identifying the most influential variables, permutation-based feature importance was utilized [36]. This approach measures a feature (variable) importance by calculating the increase of the model's prediction error after permuting the feature.

We reported partial dependence plots (PDPs) using the "pdp" package in R to understand the marginal effect of a feature on the predicted outcome. PDP demonstrates how the response variable changes as we change the value of a feature while taking into account the average effect of all the other features in the model [37]. The Y axis shows how the predicted value changes with change in predictor variables. If the line in the plot is constant near zero it means that the variable has no effect on the model. A negative value means that a specific value of the predictor variable is less likely to predict the correct class of outcome, whereas a positive value means the predictor variable has a positive impact on predicting the correct class [38]. A LOESS (locally estimated scatterplot smoothing) line is fit to show the trend.

Results

Of 4,510 patients (51.2% females, and 68.5±8.88 years) who were tested, 29.4% were positive. Females were less likely to be positive (males: 31.6% versus females: 27.3%, p=0.001).

For peer review only - http://bmjopen.bmj.com/site/about/guidelines.xhtml

In descriptive analyses, there was a difference in age between test-positive and test-negative individuals (p<0.001); specifically, those younger than 60 years (Test positive vs negative: 28% vs 21.8%) and those older than 80 years (Test + vs -: 6.2% vs 5.7%). Similarly, there was significant difference in test positivity amongst ethnicities (minority ethnicity: Test + vs -: 13.2% vs 7.6% p<0.001), and individuals born outside of the UK (Test + vs -: 14.3% vs 9.8%, p<0.001). Moreover, infected patients had a higher BMI (Test + vs - : 28.9±5.4 vs 28.2±5.4, p <0.001) ,WHR (Test + vs - : 0.89±0.09 vs 0.88±0.09), HbA1C (Test + vs - : 37.8±9.1 vs 37.1±8.3, p=0.03), LDL to HDL ratio (Test + vs - : 2.7±0.8 vs 2.5±0.9, p<0.001) , and lower HDL (Test + vs - : 1.3±0.3 vs 1.4±0.3, p<0.001) levels. On the other hand, smokers (Test + vs - : 11% vs 14%,P=0.02), alcohol drinkers (Test + vs - : 15.8% vs 20.8%, p<0.001), more educated individuals (Test + vs - : 31.2% vs 35.9%, p<0.001), house owners (Test + vs - : 79.6% vs 82.2%, p=0.04), and those who did not participate in any social activity (Test + vs - : 31.8% vs 32.7%, p=0.003) had a lower rate of infection. Test positive patients tended to have a greater number of people in their household (Test + vs -: 2.5±1.3 vs 2.3±1.5, p<0.001) and live in more deprived areas (p<0.001). These results held true in a sex-stratified analysis (Tables 1 and 2).

For peer review only

Table 1. Descriptive characteristics of COVID-19 test positive versus negative males and females

Baseline Characteristics	Overall				Females			Males		
	All	Test -	Test +	P	Test -	Test +	P	Test -	Test +	P
	N=4510	N=3184	N=1326		N=1679	N=630		N=1505	N=696	
**Demographics**										
**Age Group, %**										
<60 years	23.6	21.8	28	<0.001*	26.4	34.1	0.001*	16.5	22.4	0.006
>=60 - <70	24.7	25.4	23		26.6	25.6		24	20.7	
>=70 -<80	45.9	47.1	42.8		42	35.1		52.9	49.9	
>=80	5.9	5.7	6.2		4.9	5.2		6.6	7	
**Sex, %**										
Female	51.2	52.7	47.5	0.001*						
**Ethnicity, %**										
White	90.7	92.4	86.8	<0.001*	91.5	86.4	<0.001*	93.4	87.1	<0.001*
Mixed	2.3	2.2	2.4		2.6	2.2		1.7	2.6	
Asian	3.3	2.6	5		2.1	5.3		3.1	4.8	
Black British	3.7	2.9	5.8		3.8	6.1		1.9	5.5	
**Country of Birth, %**										
United Kingdom	88.9	90.2	85.7	<0.001*	89.1	84	0.001*	91.5	87.4	0.003
**Biological Variables**										
**Number of Medications**										
	3.3±3.3	3.3±3.3	3.2±3.3	0.7	3.2±3.2	3.2±3.3	0.8	3.3±3.4	3.3±3.3	0.6
**BMI (Kg/m2)**										
	28.4±5.4	28.2±5.4	28.9±5.4	<0.001*	28.1±5.8	28.7±6.2	0.02	28.3±4.9	29.1±4.7	<0.001*
**WHR**										
	0.9±0.1	0.8±0.09	0.8±0.09	<0.001*	0.8±0.07	0.8±0.07	0.03	0.9	0.9	0.1
**HTN, %**										
	35.3	34.5	37	0.1	28.8	31.7	0.1	41	41.7	0.7
**Stroke, %**										
	3	3	3	1	1.7	2.1	0.6	4.4	3.8	0.5
**Heart Attack, %**										
	4	4.1	3.8	0.7	1.4	1.8	0.6	7.1	5.7	0.2
**Angina, %**										
	5.7	5.9	5.3	0.4	3.3	3.4	1	8.8	7.1	0.2
**DVT, %**										
	3.3	3.1	4	0.1	2.9	4.1	0.1	3.3	3.8	0.6
**PE, %**										
	1.7	1.9	1.1	0.09	1.9	1.4	0.6	1.9	0.9	0.09
**COPD, %**										
	3.2	3.2	3.1	0.9	2.6	2.6	1	3.9	3.6	0.8
**Asthma, %**										
	13.5	13.6	13	0.5	16.4	16.3	0.9	10.5	10	0.7
**Allergy, %**										
	21.9	22.8	19.8	0.03	26.2	22.6	0.08	19	17.3	0.3
**Diabetes, %**										
	9.8	9.6	10.3	0.5	7.4	8	0.7	12	12.4	0.8
**Cancer, %**										
	9.4	10.1	7.7	0.01	11.1	9.2	0.2	9.1	6.4	0.4
**Disability, %**										

For peer review only - http://bmjopen.bmj.com/site/about/guidelines.xhtml

	45.6	46.1	44.3	0.2	42.2	38.9	0.1	50.3	49.2	0.6
**Vitamin D level (nmol/L)**										
	46.5±21.2	46.6±21	46.1±21.6	0.4	45.7±20.7	45.2±20.9	0.6	47.6±21.4	46.7±22.2	0.4
**HbA1C level (mmol/mol)**										
	37.3±8.5	37.1±8.3	37.8±9.1	0.03	36.6±7.7	36.7±8.1	0.7	37.7±8.9	38.7±9.8	0.02
**LDL level (mmol/l)**										
	3.4±0.8	3.4±0.8	3.4±0.8	0.7	3.4±0.8	3.5±0.8	0.5	3.3±0.8	3.3±0.8	0.8
**HDL level (mmol/l)**										
	1.4±0.3	1.4±0.3	1.3±0.3	<0.001*	1.5±0.3	1.4±0.3	<0.001*	1.3±0.3	1.2±0.2	0.004
**LDL to HDL Ratio**										
	2.6±0.9	2.5±0.9	2.7±0.8	<0.001*	2.3±0.8	2.5±0.8	<0.001*	2.8±0.9	2.8±0.8	0.3

Results are presented as mean ± standard deviation for numerical variables and percentage for categorical variables.
BMI: Body Mass Index, WHR: Waist to Hip Ratio, HTN: Hypertension, DVT: Deep Vein Thrombosis, PE: Pulmonary Embolism, COPD: Chronic Obstructive Pulmonary Disease, HbA1c: Hemoglobin A1c, LDL: Low Density Lipoprotein, HDL: High Density Lipoprotein
****Bonferroni Correction=0.05/24 (number of variables) =0.002, Based on this correction the differences marked with * are considered statistically significant***

Table 2. Gender variables in patients who tested positive compared to test-negative patients in overall, males and females

Gender Variables,	Overall				Female			Male		
	**All**	**Test -**	**Test +**	**P**	**Test -**	**Test +**	**P**	**Test -**	**Test +**	**P**
	**N=4474**	**N=3161**	**N=1313**		**N=1668**	**N=625**		**N=1493**	**N=688**	
**Behavioral/Lifestyle Risk Factors**										
**Smoking, %**										
0=Never	48.5	48.3	49	0.02	54.5	58.7	0.1	41.3	40.1	<0.001*
1=Previous	38.4	37.8	40		34.5	30.9		41.5	48.3	
2=Current	13.1	14	11		11	10.4		17.2	11.6	
**Alcohol, %**										
0=never	11.6	10.9	13.3	<0.001*	11.8	16.4	0.001*	9.9	10.6	0.002*
1=occasionally	14.1	14.1	14.2		18.4	18.3		9.3	10.4	
2=1 o 3 times/month	11.8	11	13.9		14	16.9		7.7	11.1	
3=1-2/week	24.1	23.6	25.4		24.1	23.8		23.1	26.8	
4=3-4/week	19	19.6	17.4		16.2	14.1		23.4	20.4	
5=daily/almost daily	19.4	20.8	15.8		15.6	10.5		26.7	20.7	
**Gender Related Variables**										
**Employment Status, %**										
Employed	50.4	49.6	52.4	0.09	53.7	58.6	0.04	45	46.7	0.5
**Night Shift, %**										
0: Never/rarely	45.1	47.5	41.4	0.21	49	40.9	0.17	45.6	42	0.8
1: Sometimes	32.1	31.6	33		31.5	32.5		31.7	33.6	
2: Usually/Always	22.8	20.9	25.6		19.5	26.6		22.8	24.4	
**Number of Children**										
	1.9±1.4	1.8±1.4	1.9±1.4	0.03	1.8±1.2	1.9±1.2	0.15	1.8±1.5	1.9±1.6	0.12
**Education, %**										
None/	36.4	34.4	41.3	<0.001*	31.2	37.5	0.02	38	44.8	0.02
NVQ/HND/HNC/CS	19.2	19.8	17.7		20.8	19.2		18.6	16.3	
Es	9.9	9.9	9.8		10.5	10.6		9.2	9.1	

For peer review only - http://bmjopen.bmj.com/site/about/guidelines.xhtml

O-Level/ GCSEs<br>A-Levels/AS-Levels<br>College/University/<br>Professional	34.5	35.9	31.2		37.5	32.6		34.2	29.8	
**Deprivation indices:**										
Education Score										
	18.6±17.9	17.7±17.4	20.6±19.3	<0.001*	17.6±17.1	20.9±19.3	<0.001*	17.9±17.6	20.4±18.9	0.003
Environmental Score										
	20.6±16.4	20±15.7	22.1±17.8	<0.001*	19.9±15.6	21.3±17.9	0.08	20.1±15.9	22.7±17.6	0.001*
Employment Score										
	0.1±0.06	0.09±0.06	0.1±0.07	<0.001*	0.09±0.06	0.1±0.07	<0.001*	0.10±0.06	0.11±0.07	0.01
Health Score										
	0.09±0.9	0.05±0.9	0.2±0.8	<0.001*	0.05±0.8	0.1±0.9	0.02	0.06±0.9	0.2±0.08	<0.001*
Housing Score										
	19.7±10.2	19.8±10.3	19.7±9.9	0.7	19.8±10.2	19.9±9.9	0.7	19.8±10.5	19.4±9.9	0.4
Income Score										
	0.1±0.11	0.1±0.1	0.1±0.11	<0.001*	0.1±0.1	0.1±0.12	<0.001*	0.1±0.11	0.1±0.11	0.008
**Income, %**										
1= <18K<br>2=18-30.9K<br>3=31-51.9K<br>4=52-100K<br>5=>100K	32<br>24.6<br>22.3<br>16.5<br>4.6	31.5<br>24.5<br>22.2<br>16.7<br>5.1	33.5<br>24.6<br>22.6<br>16<br>3.3	0.1	31.2<br>23.3<br>23.2<br>17.5<br>4.8	32.1<br>24<br>26.5<br>14.3<br>3.2	0.17	31.8<br>25.8<br>21.1<br>16<br>5.3	34.7<br>25.2<br>19.1<br>17.5<br>3.5	0.26
**Number in Household**										
	2.4±1.5	2.3±1.5	2.5±1.3	0.001*	2.4±1.8	2.5±1.4	0.04	2.3±1.1	2.4±1.3	0.006
**House Ownership, %**										
	81.4	82.2	79.6	0.04	84.5	80.4	0.02	79.6	78.8	0.7
**Neuroticism Score**										
	4.3±3.3	4.3±3.3	4.2±3.3	0.4	4.8±3.3	4.8±3.2	0.9	3.8±3.2	1.7±3.3	0.5
**Risk Taking Behavior, %**										
	30.7	29.9	32.5	0.1	23.3	27	0.7	37.3	37.5	0.9
**Leisure/Social Activities, %**										
0=No<br>1=Sports Club or Gym<br>2=Pub or Social Club<br>3=Religious Group<br>4=Adult Education Class<br>5= Other Group Activity	32.4<br>24.1<br>21.1<br>10.4<br>2.8<br>9.1	32.7<br>24.6<br>20.4<br>9.5<br>3.1<br>9.7	31.8<br>23<br>22.6<br>12.5<br>2.2<br>7.8	0.003	32.9<br>25.8<br>13.6<br>11.5<br>4.4<br>11.8	34.3<br>21.9<br>16.1<br>14.8<br>3.7<br>9.3	0.03	32.4<br>23.2<br>28<br>7.3<br>1.5<br>7.5	29.6<br>24.1<br>28.6<br>10.4<br>0.9<br>6.4	0.09

Results are presented as mean ± standard deviation for numerical variables and percentage for categorical variables.

****Bonferroni Correction=0.05/18 (number of variables) =0.003, Based on this correction the differences marked with * are considered statistically significant***

Machine Learning-based Prediction Models for SARS-Cov-2 Positive Test

The area under curve (AUC) for test positivity in the overall model, male and female specific models were 0.570 (95% confidence interval (CI): 0.537-0.604), 0.575 (0.529-0.621), 0.561 (95% CI: 0.512-0.609), respectively. The performance of the gradient boosted decision tree models is summarized in Table 3. Figure 1-3 illustrate the order of variable importance and partial dependence plots used for interpreting the results and direction of each variable in the models. The prediction models for the overall study population suggest that an increased LDL to HDL ratio, WHR, and age were associated with a higher likelihood of test positivity. Additionally, individuals who worked night shifts or lived in a more deprived area (lower environment and education scores) as well as those participating in social activities—including sports clubs, bars, and religious groups—had a higher risk of having a positive test. In contrast, individuals with higher education levels, higher income, and those with daily or almost daily alcohol consumption were less likely to have a positive result.

The sex-specific models for test positivity showed that gender factors were more important in females, whereas in males, biological factors were significant contributors to test positivity. Females who lived in more deprived areas (increased environment score), had increased LDL to HDL ratio, worked night shifts, and had a greater number of family members in their household were more likely to test positive. Moreover, those with income greater than 100K were less likely to test positive. (Figure 2). In comparison, males with greater BMI and LDL to HDL ratio, more deprived area (greater score) and Black British ethnicity were more likely to test positive (Figure 3).

For peer review only - http://bmjopen.bmj.com/site/about/guidelines.xhtml

Table 3. Summary of GBM model performance for predicting test-positive results in training (10-fold cross-validation) and test sets

		Accuracy	AUC	Threshold	PRAUC	Precision	Recall	Specificity
**Overall**	Test Set	0.56	0.570 (0.537-0.604)	0.25	0.33	0.34	0.56	0.56
**Male**	Test Set	0.53	0.575 (0.529-0.621)	0.32	0.38	0.37	0.67	0.46
**Female**	Test Set	0.62	0.561 (0.512-0.609)	0.26	0.31	0.32	0.35	0.72

**Area Under Curve (AUC) is reported with 95% Confidence Interval (CI)*
*** Used the best threshold of ROC as cut off for determining precision/recall, sensitivity/specificity*

For peer review only

ML-based Prediction Models for COVID-19 Related Hospitalizations

The area under curve (AUC) for hospitalization in test positive patients in overall, male, and female specific models were 0.60 (95% CI: 0.534-0.665)), 0.544 (95% CI: 0.453-0.635), and 0.612 (95% CI: 0.532-0.692), respectively. The performance of the gradient boosted decision tree models is summarized in Table 4. Amongst the 1,326 test-positive patients, 932 (70.3%) were hospitalized (females: 413 (44.3%)). Figure 4-6 illustrate the order of variable importance and partial dependence plots used for interpreting the results and direction of each variable in the models. The result of the overall model to predict hospitalization in test-positive patients revealed that those with higher HbA1c level, older age, greater BMI, higher LDL to HDL ratio and greater number of medications had greater risk of being hospitalized (Figure 4).

The sex stratified model revealed that older age, a higher level of HbA1c, LDL to HDL ratio, a greater number of medications and higher-housing score (showing more deprived areas) were most influential variables in predicting hospitalization in test-positive females (Figure 5), whereas older age, an increased HbA1c level, WHR, LDL to HDL ratio and BMI were the most

influential variables associated with hospitalization in test-positive males (Figure 6).

Table4. Summary of GBM model performance for predicting being hospitalized in test-positive patients in training (10-fold cross-validation) and test sets

		Accuracy	AUC	Threshold	PRAUC	Precision	Recall	Specificity
**Overall**	Test Set	0.61	0.60 (0.534-0.665)	0.69	0.75	0.77	0.66	0.48
**Male**	Test Set	0.51	0.544 (0.453-0.635)	0.75	0.76	0.85	0.43	0.75
**Female**	Test Set	0.55	0.612 (0.532-0.692)	0.68	0.75	0.81	0.47	0.75

**Area Under Curve (AUC) is reported with 95% Confidence Interval (CI)*
*** Used the best threshold of ROC as cut off for determining precision/recall, sensitivity/specificity*

Discussion

The present evaluation of individuals tested for SARS-CoV-2 in a UK cohort demonstrates the importance of gender-related factors along with clinical characteristics in predicting COVID-19 test positivity, hence providing guidance for identifying vaccination priority groups in the general population. Whilst factors related to the gender role of individuals were the most influential determinants in females, cardio-metabolic risk factors played a key role in males. Such a sex-specific cluster of factors associated with adverse outcomes was attenuated when considering the rate of COVID-19 related hospitalization among test-positive individuals. Notably, older age and cardio-metabolic diseases, including diabetes, obesity, and dyslipidemia, were most influential regardless of sex.

Emerging evidence has shown sex differences in contracting and severity of the infection. While most investigations have focused on biological factors as the potential culprit, few have incorporated gender determinants. Various modulating mechanisms have been suggested, including genetic factors (hormone-regulated expression of genes), the difference in innate and

For peer review only - http://bmjopen.bmj.com/site/about/guidelines.xhtml
For peer review only

adaptive immune responses, as well as gendered factors such as lifestyle, behavioral and psychosocial factors [11 39 13].

Our findings reinforce and advance the current evidence related to the impact of metabolic comorbidities and older age in the COVID-19 pandemic. Obesity has been linked with impaired pulmonary function and suppression of immune response and has been recognized as one of the most important factors in contracting COVID-19 infection, the severity of the disease, and mortality [4 40]. Higher BMI and WHR are some of the more influential measures that have shown a dose-response relationship with test positivity and disease severity [4 6 40]. A study on the UK Biobank data demonstrated a more than 50 percent increase in COVID-19 infection in obese and severely obese patients compared to non-obese individuals [40]. Investigation of different databases in various countries have also reported older age and comorbidities as the most important factors associated with clinical severity and mortality [41]. Specifically, diabetes has been associated with more severe disease manifestation [42]. This is consistent with our results, which demonstrated HbA1c level as one of the most prominent factors for hospitalization in the overall, and sex-stratified models. Preliminary studies have demonstrated the association between HDL level, infection, and disease severity. While the mechanism of this correlation is unknown, this can be due to the antioxidant, antithrombotic and anti-inflammatory role of HDL cholesterol [43].

A major and novel contribution of this study is the application of a sex and gender-oriented lens to inform the understanding of COVID-19 infection by conducting sex-disaggregated analyses and incorporating both sex and gender-related determinants. Studies have shown socioeconomic disadvantages such as living in a more deprived area and lower education to be associated with increased risk of infection and disease severity [2 4]; these factors prevail in

For peer review only - http://bmjopen.bmj.com/site/about/guidelines.xhtml

individuals more likely to work in service-based occupations, be self-employed, or live in crowded households [3 4 44]. Moreover, less access to healthcare is another factor that leads to greater infection risk and worst outcomes. By the same token, our study demonstrated that those who live in a more deprived area with lower SES and education were more susceptible to COVID-19 infection, but the impact of gender determinants was more significant amongst females. Women's role as caregivers within family and society increases their risk of infection [39]. Moreover, women are more likely to work as frontline workers, including nursing positions, increasing their exposure to the virus [3 22 39]. In our current study, environment score, household arrangement, working night shifts, and income were amongst the most important factors for females. In contrast, obesity, LDL to HDL ratio, and alcohol consumption were amongst the most influential factors in men.

The results of this study serve as an important guide for vaccination prioritization policies. While essential workers and elderly individuals have already been targeted for vaccination, the next step will be the identification and prompt vaccination of individuals in higher-risk groups in the general population. Although factors such as diabetes and obesity might be important, psychosocial risk factors such as lower socioeconomic status, education level, living in a more deprived environment, risky occupation, and household crowding should also be taken into account. Individuals, especially females, exhibiting such high-risk gendered factors should be prioritized for vaccination.

Strengths and Limitations

A unique feature of the study is the investigation of numerous lifestyles, socioeconomic, mental, and behavioral factors representing different dimensions of gender in conjunction with clinical and laboratory factors made feasible through machine learning algorithms. Furthermore,

For peer review only - http://bmjopen.bmj.com/site/about/guidelines.xhtml

the assessment of sex-stratified algorithms to elucidate the most influential factors in both sexes adds novel information as it has rarely been done to date.

The study should also be interpreted considering some limitations. The first limitation of this study is selection bias due to the lack of systematic and random testing across the UK. Second, this analysis was done using the baseline data that was collected from 2006 to 2010 and not the ones done at the time of COVID infection diagnosis. Therefore, misclassification of determinants is possible. However, previous studies of the UK Biobank have shown a high correlation between baseline and follow-up data for a subsample of patients who had further visits for imaging [22]. Moreover, a disproportionately higher representation of Caucasians in the study may make the results less generalizable to other ethnic groups. Finally, though we attempted to test various clinical, social and demographic factors to predict test positivity, it is essential to note that acquisition of infection is a multifactorial phenomenon that cannot be easily encoded using a small set of variables. Our relatively low predictive model performance—as expected—reflects other influences not captured in the UK Biobank. Similar results were obtained using the XGboost method from a recent study on the UK Biobank dataset whereby slightly superior performance to our gbm model was obtained, which further supports the interpretation that this is the expected accuracy for predictive models on this dataset[18]. While, similar features were obtained for predicting severity (hospitalization and fatality) in this study, combining mortality with hospitalization for assessing severity can justify better model performance compared to only hospitalization in our models. The difference observed in the performance of our model compared to the aforementioned study maybe also explained by the lower power and heterogeneity in our sample. Moreover, since the predictive accuracy of the model is slightly low, the risk factors deduced may not be strong enough to predict the outcomes.

For peer review only - http://bmjopen.bmj.com/site/about/guidelines.xhtml

Additionally with the emergence of the dominant delta and omicron variant further studies are needed to elucidate the risk factors, though we suspect them to remain the same. Finally, the small sample size for this analysis may limit generalizability.

Conclusions

Sex-specific risk patterns of COVID-19 test-positivity exist, with gender-related factors being more relevant in females and biological factors in males. Specifically, socioeconomic status, education level, number of people living in a household, and high-risk jobs were associated with a higher likelihood of contracting the infection in female individuals, whereas cardiometabolic disease and obesity were more associated in males. The rate of COVID-19 related hospitalization recognized similar favoring clinical factors regardless of sex. This study highlights the importance of prioritizing high-risk groups using psychosocial determinants along with clinical factors as a targeted approach for vaccination of more at-risk population to contain SARS-Cov-2 pandemic.

Collaborators: The GOING FWD Investigators: Alexandra Kautzky-Willer, Medical University of Vienna, Austria; **Karolina Kublickiene**: Karolinska Institutet, Sweden; **Maria Trinidad Herrero**: Universidad de Murcia, Spain

Contributors:

Drs Pilote and El-Emam contributed equally as co-senior authors. ZA, YS, PA, CN, VR, LP, KEE and GOING FWD Investigators designed the study, developed the questions and contributed to the development of conceptual framework and writing the first draft. ZA, YS, and KEE performed the main analysis and critical revisions of manuscript. FM and RF validated the results by performing secondary analyses and finalizing the draft. ZA, YS, PA, CN, VR, LP, KEE and GOING FWD Investigators supervised the project from study design to final draft and approved

For peer review only - http://bmjopen.bmj.com/site/about/guidelines.xhtml

the final version of manuscript. All Authors agree to be accountable for all aspects of the work ensuring that questions related to the accuracy or integrity of any part of the work are appropriately investigated and resolved.

Funding: The GOING-FWD Consortium is funded by the GENDER-NET Plus ERA-NET Initiative (Project Ref. Number: GNP-78): The Canadian Institutes of Health Research (GNP-161904), "La Caixa" Foundation (ID 100010434) with code LCF/PR/DE18/52010001, The Swedish Research Council (2018-00932) and The Austrian Science Fund (FWF, I 4209). VR was funded by the Scientific Independence of Young Researcher Program of the Italian Ministry of University, Education, and Research (RBSI14HNVT).

Patient and Public Involvement: No patient involved in the design, or conduct, or reporting, or dissemination plans of our research.

Patient consent for publication: Not applicable

Ethics approval: Ethics approval for the project was obtained from the coordinator center at McGill University, Canada (REB # 2020-5452 (Gender Outcomes INternational Group: to Further Well-being Development (GOING-FWD), CIHR GNP-161904, 2018/10/01 to 2023/03/31). The data was obtained from the UK Biobank repository (Application ID:45551).

Competing interests: There are no competing interests for any author. KEE is a co-founder and has equity in Replica Analytics Ltd., which is a spin-off from the Children's Hospital of Eastern Ontario Research Institute and the University of Ottawa developing synthetic data generation software.

Data availability statement: No additional data available. The data can be obtained by registering at https://www.ukbiobank.ac.uk/.

Disclosures:

The authors disclose no conflict of interest related to this study.

For peer review only - http://bmjopen.bmj.com/site/about/guidelines.xhtml

Figure Legends:

Figure 1: Overall Results: Partial dependency plots for predicting test positive results for first 10 most influential variables using permutation methods in the overall population using Shrinkage=0.01, bag fraction=0.5, interaction depth=5, CV fold=10, train fraction=0.7, n.minobsinnode = 10 as hyperparameters from grid search.

*The X axis is the predictor variable in the model. The Y axis shows how the predicted value changes with change in predictor variables. If the line in the plot is constant near zero it means that the variable has no effect on the model. A negative value means that a specific value of the predictor variable is less likely to predict the correct class of outcome, whereas a positive value means the predictor variable has a positive impact on predicting the correct class. A LOESS (locally estimated scatterplot smoothing) line is fit to show the trend.

** The number beside each variable shows the order of feature importance and most influential variables for each model. For identifying the most influential variables, permutation-based feature importance was utilized. This approach measures a feature (variable) importance by calculating the increase of the model's prediction error after permuting the feature.

Figure 2: Results for Females: Partial dependency plots for predicting test positive results for first 5 most influential variables using permutation methods in Females using Shrinkage=0.01, bag fraction=1, interaction depth=7, CV fold=10, train fraction=0.8, n.minobsinnode = 15 as hyperparameters from grid search.

Figure 3: Results for Males: Partial dependency plots for predicting test positive results for first 5 most influential variables using permutation methods in males using, Shrinkage=0.001, bag fraction=1, interaction depth=3, CV fold=10, train fraction=0.8, n.minobsinnode = 5 as hyperparameters from grid search

Figure 4: Hospitalization in overall population: Partial dependency plots for predicting hospitalization in test positive patients for first 5 most influential variables using permutation methods using, Shrinkage=0.1, bag fraction=0.8, interaction depth=3, CV fold=10, train fraction=0.7, n.minobsinnode = 15 as hyperparameters from grid search

Figure 5: Hospitalization of females: Partial dependency plot for predicting hospitalization in test positive females for first 5 most influential variables using permutation methods using, Shrinkage=0.05, bag fraction=0.65, interaction depth=7, CV fold=10, train fraction=0.8, n.minobsinnode = 15 as hyperparameters from grid search

Figure 6: Hospitalization of males: Partial dependency plot for predicting hospitalized patients in test positive males for first 5 most influential variables using permutation methods using,

For peer review only - http://bmjopen.bmj.com/site/about/guidelines.xhtml

Shrinkage=0.1, bag fraction=0.5, interaction depth=5, CV fold=10, train fraction=0.8, n.minobsinnode = 15 as hyperparameters from grid search

References:

1. Johns Hopkins University J. COVID-19 Dashboard by the Center for Systems Science and Engineering (CSSE) 2021 [updated 11/21/2021. Available from: https://www.arcgis.com/apps/opsdashboard/index.html#/bda7594740fd40299423467b48e9ecf6.
2. Niedzwiedz CL, O'Donnell CA, Jani BD, et al. Ethnic and socioeconomic differences in SARS-CoV-2 infection: prospective cohort study using UK Biobank. BMC Medicine 2020;**18**(1):1-14.
3. Lassale C, Gaye B, Hamer M, et al. Ethnic Disparities in Hospitalization for COVID-19: a Community-Based Cohort Study in the UK. Brain Behav Immun 2020;**88**:44-49.
4. Prats-Uribe A, Paredes R, Prieto-Alhambra D. Ethnicity, comorbidity, socioeconomic status, and their associations with COVID-19 infection in England: a cohort analysis of UK Biobank data. medRxiv 2020.
5. Kolin DA, Kulm S, Elemento O. Clinical and Genetic Characteristics of Covid-19 Patients from UK Biobank. Plos one 2020;**15**(11):e0241264.
6. Hamer M, Kivimäki M, Gale CR, et al. Lifestyle Risk Factors, Inflammatory Mechanisms, and COVID-19 Hospitalization: A Community-Based Cohort Study of 387,109 Adults in UK. Brain, Behavior, and Immunity 2020;**87**:184-7.
7. Patel AP, Paranjpe MD, Kathiresan NP, et al. Race, Socioeconomic Deprivation, and Hospitalization for COVID-19 in English participants of a National Biobank. International journal for equity in health 2020;**19**(1):1-4.
8. Darling AL, Ahmadi KR, Ward KA, et al. Vitamin D status, body mass index, ethnicity and COVID-19: Initial analysis of the first-reported UK Biobank COVID-19 positive cases (n 580) compared with negative controls (n 723). Proceedings of the Nutrition Society 2020;**80**(OCE1):E17.
9. Schiffer VM, Janssen EB, van Bussel BC, et al. The" sex gap" in COVID-19 trials: a scoping review. EClinicalMedicine 2020;**29-30**:100652.
10. Tadiri CP, Gisinger T, Kautzy-Willer A, et al. The influence of sex and gender domains on COVID-19 cases and mortality. CMAJ 2020;**192**(36):E1041-E45.
11. Scully EP, Haverfield J, Ursin RL, et al. Considering how biological sex impacts immune responses and COVID-19 outcomes. Nature Reviews Immunology 2020;**20**:442–47.
12. Galbadage T, Peterson BM, Awada J, et al. Systematic Review and Meta-Analysis of Sex-Specific COVID-19 Clinical Outcomes. Front Med (Lausanne) 2020;**23**(7):348.
13. Haitao T, Vermunt JV, Abeykoon J, et al. COVID-19 and Sex Differences: Mechanisms and Biomarkers. Mayo Clinic proceedings 2020;**95**(10):2189-203.
14. Johnson JL, Greaves L, Repta R. Better science with sex and gender: Facilitating the use of a sex and gender-based analysis in health research. International Journal for Equity in Health 2009;**8**(1):1-11.
15. CIHR OTM. Online Training Modules: Integrating Sex & Gender in Health Research 2021 [updated 12/07/2020. Available from: https://cihr-irsc.gc.ca/e/49347.html.
16. World Health Organization (WHO) S. Addressing sex and gender in epidemic-prone infectious diseases 2007 [updated 12/07/2020. Available from: https://www.who.int/csr/resources/publications/SexGenderInfectDis.pdf.
17. Al Youha S, Alkhamis M, Al Mazeedi S, et al. Using Machine Learning to Unveil Demographic and Clinical Features of COVID-19 Symptomatic and Asymptomatic Patients. 2020.
18. Wong KC, Xiang Y, Yin L, et al. Uncovering clinical risk factors and prediction of severe COVID-19: A machine learning approach based on UK Biobank data. JMIR Public Health Surveill 2021;**7**(8):e29544.
19. Ahamad MM, Aktar S, Rashed-Al-Mahfuz M, et al. A machine learning model to identify early stage symptoms of

For peer review only - http://bmjopen.bmj.com/site/about/guidelines.xhtml

SARS-Cov-2 infected patients. Expert systems with applications 2020;**160**:113661.
20. Sudlow C, Gallacher J, Allen N, et al. UK biobank: an open access resource for identifying the causes of a wide range of complex diseases of middle and old age. Plos med 2015;**12**(3):e1001779.
21. Armstrong J, Rudkin JK, Allen N, et al. Dynamic linkage of covid-19 test results between public health england's second generation surveillance system and uk biobank. Microbial genomics 2020;**6**(7).
22. Mutambudzi M, Niedzwiedz CL, Macdonald EB, et al. Occupation and risk of COVID-19: prospective cohort study of 120,621 UK Biobank participants. Occup Environ Med 2020;**78**(5):307–14.
23. Pilote L, Norris CM, Raparelli V, et al. Gender Outcomes INternational Group: to Further Well-being Development (GOING-FWD): https://www.mcgill.ca/going-fwd4gender/, 2020.
24. Tadiri CP, Raparelli V, Abrahamowicz M, et al. Methods for prospectively incorporating gender into health sciences research. J Clin Epidemiol 2021;**129**:191-97.
25. Cho ER, Slutsky AS, Jha P. Smoking and the risk of COVID-19 infection in the UK Biobank Prospective Study. medRxiv 2020.
26. Hastie CE, Mackay DF, Ho F, et al. Vitamin D concentrations and COVID-19 infection in UK Biobank. Diabetes Metab Syndr 2020;**14**(5):1315-16.
27. Khawaja AP, Warwick AN, Hysi PG, et al. Associations with covid-19 hospitalisation amongst 406,793 adults: the UK Biobank prospective cohort study. medRxiv 2020.
28. Friedman JH. Greedy function approximation: a gradient boosting machine. Annals of statistics 2001:1189-232.
29. Ridgeway G. Generalized Boosted Models: A guide to the gbm package. Update 2007;**1**(1):2007.
30. Bühlmann P, Hothorn T. Boosting algorithms: Regularization, prediction and model fitting. Statistical Science 2007;**22**(4):477-505.
31. Maleki F, Ovens K, Najafian K, et al. Overview of Machine Learning Part 1: Fundamentals and Classic Approaches. Neuroimaging Clin N Am 2020;**30**(4):e17-e32.
32. Maleki F, Muthukrishnan N, Ovens K, et al. Machine Learning Algorithm Validation: From Essentials to Advanced Applications and Implications for Regulatory Certification and Deployment. Neuroimaging Clin N Am 2020;**30**(4):433-45.
33. Yan Y. MLmetrics: Machine learning evaluation metrics. R package version 2016;**1**(1).
34. Davis J, Goadrich M. The relationship between Precision-Recall and ROC curves. Proceedings of the 23rd international conference on Machine learning. Pittsburgh, Pennsylvania, USA: Association for Computing Machinery, 2006:233–40.
35. Boyd K, Costa VS, Davis J, et al. Unachievable region in precision-recall space and its effect on empirical evaluation. Proc Int Conf Mach Learn 2012;**2012**:349.
36. Nicodemus KK, Malley JD, Strobl C, et al. The behaviour of random forest permutation-based variable importance measures under predictor correlation. BMC bioinformatics 2010;**11**(1):1-13.
37. Greenwell BM. pdp: An R Package for Constructing Partial Dependence Plots. R J 2017;**9**(1):421.
38. Khoda Bakhshi A, Ahmed MM. Utilizing black-box visualization tools to interpret non-parametric real-time risk assessment models. Transportmetrica A: Transport Science 2021;**17**(4):739-65.
39. Gebhard C, Regitz-Zagrosek V, Neuhauser HK, et al. Impact of sex and gender on COVID-19 outcomes in Europe. Biology of Sex Differences 2020;**11**(1):1-13.
40. Yates T, Razieh C, Zaccardi F, et al. Obesity and risk of COVID-19: analysis of UK Biobank. Primary Care Diabetes 2020;**14**:566-67.
41. Atkins JL, Masoli JA, Delgado J, et al. Preexisting comorbidities predicting severe COVID-19 in older adults in the UK Biobank community cohort. J Gerontol A Biol Sci Med Sci 2020;**75**(11):2224-30.
42. Guo W, Li M, Dong Y, et al. Diabetes is a risk factor for the progression and prognosis of COVID-19. Diabetes/metabolism research and reviews 2020;**36**(7):e3319.
43. Scalsky RJ, Desai K, Chen Y-J, et al. Baseline Cardiometabolic Profiles and SARS-CoV-2 Risk in the UK Biobank. PLoS ONE 2020;**16**(4):e0248602.
44. Aldridge RW, Lewer D, Katikireddi SV, et al. Black, Asian and Minority Ethnic groups in England are at increased risk of death from COVID-19: indirect standardisation of NHS mortality data. Wellcome Open Research 2020;**5**(88):88.

For peer review only - http://bmjopen.bmj.com/site/about/guidelines.xhtml

For peer review only

For peer review only - http://bmjopen.bmj.com/site/about/guidelines.xhtml

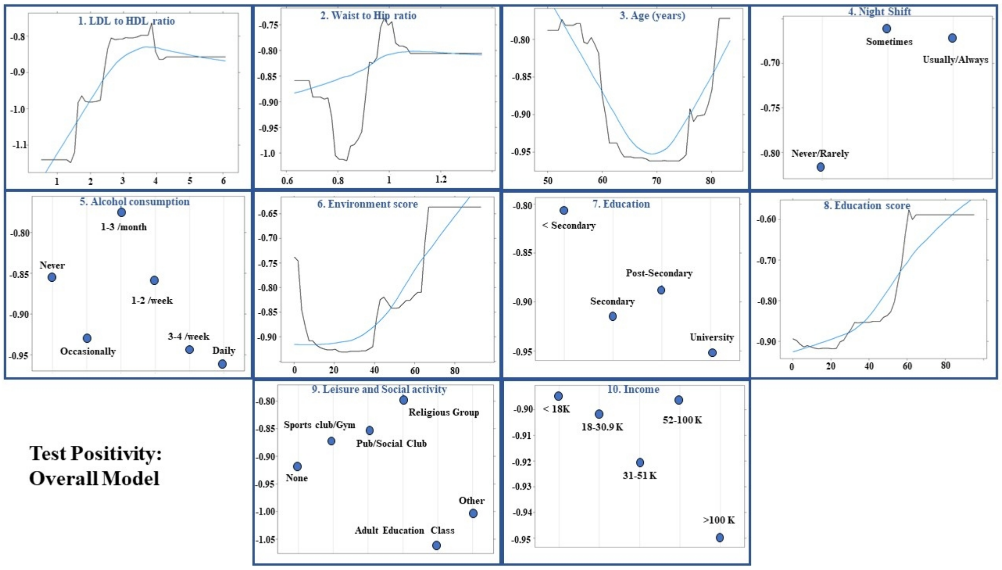

Figure 1: Overall Results: Partial dependency plots for predicting test positive results for first 10 most influential variables using permutation methods in the overall population using Shrinkage=0.01, bag fraction=0.5, interaction depth=5, CV fold=10, train fraction=0.7, n.minobsinnode = 10 as hyperparameters from grid search.

*The X axis is the predictor variable in the model. The Y axis shows how the predicted value changes with change in predictor variables. If the line in the plot is constant near zero it means that the variable has no effect on the model. A negative value means that a specific value of the predictor variable is less likely to predict the correct class of outcome, whereas a positive value means the predictor variable has a positive impact on predicting the correct class. A LOESS (locally estimated scatterplot smoothing) line is fit to show the trend.

** The number beside each variable shows the order of feature importance and most influential variables for each model. For identifying the most influential variables, permutation-based feature importance was utilized. This approach measures a feature (variable) importance by calculating the increase of the model's prediction error after permuting the feature.

338x190mm (96 x 96 DPI)

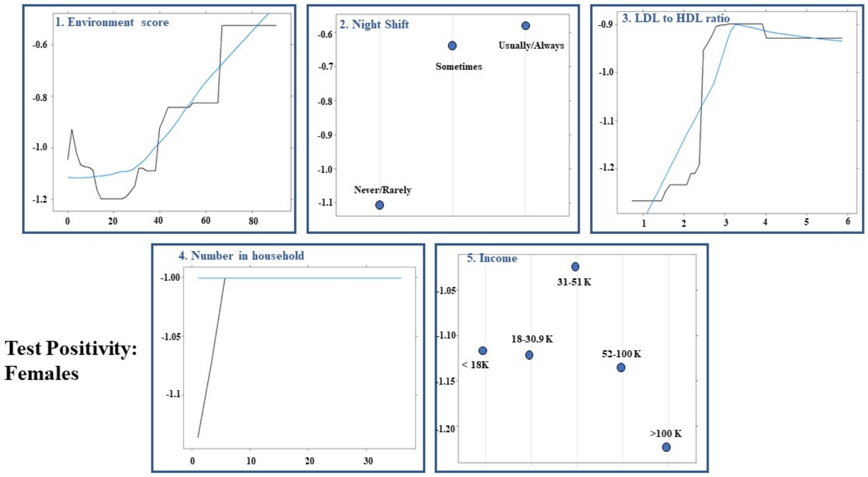

Figure 2: Results for Females: Partial dependency plots for predicting test positive results for first 5 most influential variables using permutation methods in Females using Shrinkage=0.01, bag fraction=1, interaction depth=7, CV fold=10, train fraction=0.8, n.minobsinnode = 15 as hyperparameters from grid search.

338x190mm (96 x 96 DPI)

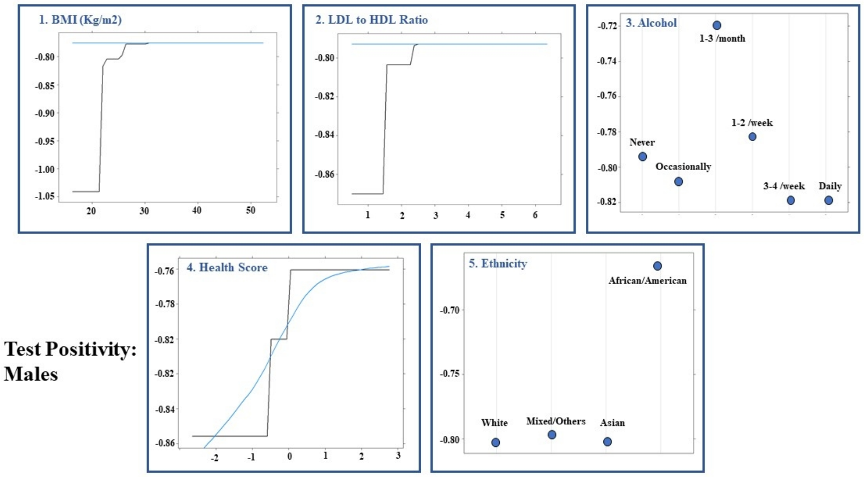

Figure 3: Results for Males: Partial dependency plots for predicting test positive results for first 5 most influential variables using permutation methods in males using, Shrinkage=0.001, bag fraction=1, interaction depth=3, CV fold=10, train fraction=0.8, n.minobsinnode = 5 as hyperparameters from grid search

338x190mm (96 x 96 DPI)

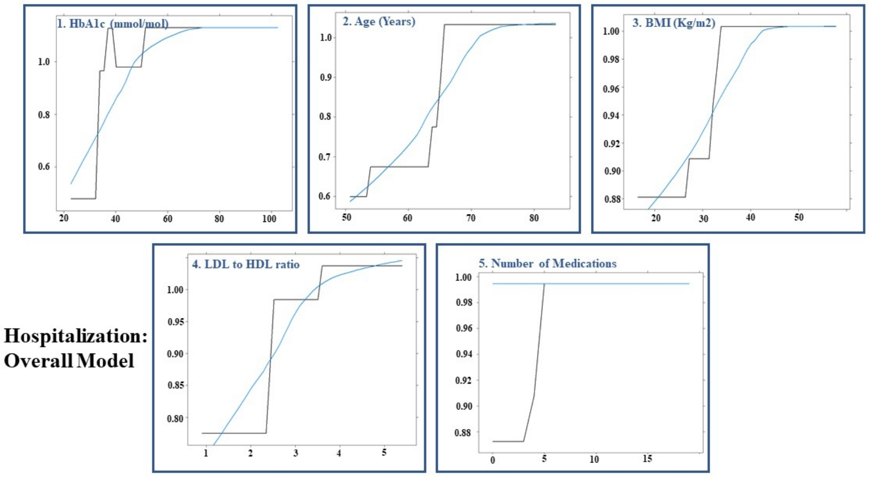

Figure 4: Hospitalization in overall population: Partial dependency plots for predicting hospitalization in test positive patients for first 5 most influential variables using permutation methods using, Shrinkage=0.1, bag fraction=0.8, interaction depth=3, CV fold=10, train fraction=0.7, n.minobsinnode = 15 as hyperparameters from grid search

338x190mm (96 x 96 DPI)

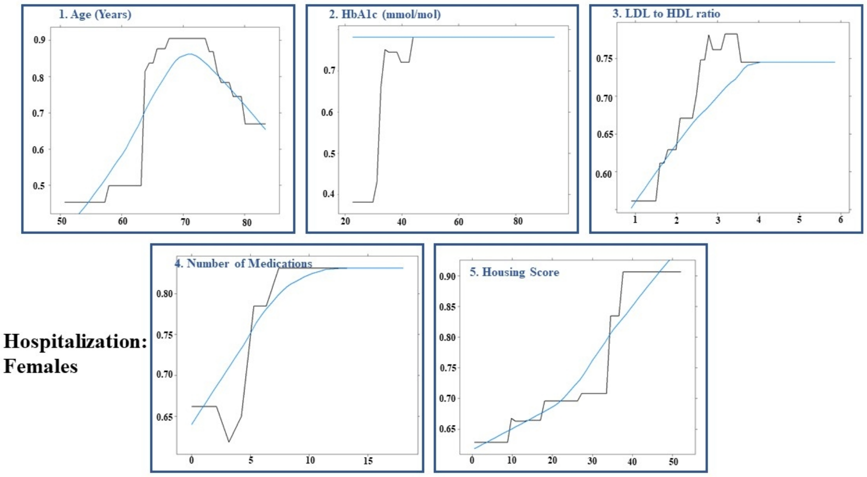

Figure 5: Hospitalization of females: Partial dependency plot for predicting hospitalization in test positive females for first 5 most influential variables using permutation methods using, Shrinkage=0.05, bag fraction=0.65, interaction depth=7, CV fold=10, train fraction=0.8, n.minobsinnode = 15 as hyperparameters from grid search

338x190mm (96 x 96 DPI)

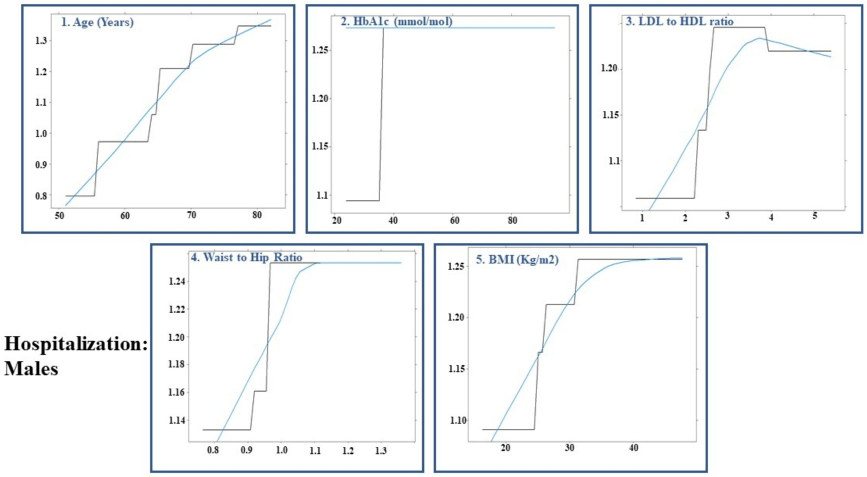

Figure 6: Hospitalization of males: Partial dependency plot for predicting hospitalized patients in test positive males for first 5 most influential variables using permutation methods using, Shrinkage=0.1, bag fraction=0.5, interaction depth=5, CV fold=10, train fraction=0.8, n.minobsinnode = 15 as hyperparameters from grid search

338x190mm (96 x 96 DPI)

Supplemental

Supplement 1. Definition of deprivation indices

Variable Name	**Definition**
Education score	This measure shows the extent of deprivation in education, skills and training in an area within 2 sub domains including children and young people and adults skills.
Living environment score	This score measures the quality of individuals' immediate surroundings both within and outside the home within the 'indoors' living environment, which measures the quality of housing, and the 'outdoors' living environment which contains two measures relating to air quality and road traffic accidents subdomains.
Employment score	This score measures employment deprivation in an area conceptualized as involuntary exclusion of the working age population from the labour market.
Income score	This score measures the proportion of the population in an area experiencing deprivation related to low income.
Housing score	This score measures the physical and financial accessibility of housing and key local services. The indicators fall into two sub-domains: 'geographical barriers', which relate to the physical proximity of local services, and 'wider barriers' which includes issues relating to access to housing such as affordability.
Health score	This score measures premature death and the impairment of quality of life by poor health. It considers both physical and mental health. The domain measures morbidity, disability and premature mortality but not aspects of behaviour or environment that may be predictive of future health deprivation.

For peer review only - http://bmjopen.bmj.com/site/about/guidelines.xhtml

Supplement 2: Grid Search Parameters

**Shrinkage**	0.3	0.1	0.05	0.01	0.001
**Interaction Depth**	3	5	7		
**N.minobsinnode**	5	10	15		
**Bag.fraction**	0.65	0.5	0.8	1	

For peer review only

STROBE 2007 (v4) checklist of items to be included in reports of observational studies in epidemiology*

Checklist for cohort, case-control, and cross-sectional studies (combined)

Section/Topic	Item #	Recommendation	Reported on page #
**Title and abstract**	1	(*a*) Indicate the study's design with a commonly used term in the title or the abstract	1-2
		(*b*) Provide in the abstract an informative and balanced summary of what was done and what was found	1-2
**Introduction**			
Background/rationale	2	Explain the scientific background and rationale for the investigation being reported	4-5
Objectives	3	State specific objectives, including any pre-specified hypotheses	4-5
**Methods**			
Study design	4	Present key elements of study design early in the paper	5
Setting	5	Describe the setting, locations, and relevant dates, including periods of recruitment, exposure, follow-up, and data collection	5
Participants	6	(*a*) *Cohort study*—Give the eligibility criteria, and the sources and methods of selection of participants. Describe methods of follow-up<br>*Case-control study*—Give the eligibility criteria, and the sources and methods of case ascertainment and control selection. Give the rationale for the choice of cases and controls<br>*Cross-sectional study*—Give the eligibility criteria, and the sources and methods of selection of participants	5
		(*b*) *Cohort study*—For matched studies, give matching criteria and number of exposed and unexposed<br>*Case-control study*—For matched studies, give matching criteria and the number of controls per case	
Variables	7	Clearly define all outcomes, exposures, predictors, potential confounders, and effect modifiers. Give diagnostic criteria, if applicable	6-7
Data sources/ measurement	8*	For each variable of interest, give sources of data and details of methods of assessment (measurement). Describe comparability of assessment methods if there is more than one group	*6-7*
Bias	9	Describe any efforts to address potential sources of bias	NA
Study size	10	Explain how the study size was arrived at	5
Quantitative variables	11	Explain how quantitative variables were handled in the analyses. If applicable, describe which groupings were chosen and why	7
Statistical methods	12	(*a*) Describe all statistical methods, including those used to control for confounding	7-8
		(*b*) Describe any methods used to examine subgroups and interactions	7-8
		(*c*) Explain how missing data were addressed	7
		(*d*) *Cohort study*—If applicable, explain how loss to follow-up was addressed<br>*Case-control study*—If applicable, explain how matching of cases and controls was addressed	

For peer review only

		Cross-sectional study—If applicable, describe analytical methods taking account of sampling strategy	
		(*e*) Describe any sensitivity analyses	7-8
**Results**			
Participants	13*	(a) Report numbers of individuals at each stage of study—eg numbers potentially eligible, examined for eligibility, confirmed eligible, included in the study, completing follow-up, and analysed	9
		(b) Give reasons for non-participation at each stage	NA
		(c) Consider use of a flow diagram	NA
Descriptive data	14*	(a) Give characteristics of study participants (eg demographic, clinical, social) and information on exposures and potential confounders	9
		(b) Indicate number of participants with missing data for each variable of interest	-
		(c) *Cohort study*—Summarise follow-up time (eg, average and total amount)	-
Outcome data	15*	*Cohort study*—Report numbers of outcome events or summary measures over time	-
		Case-control study—Report numbers in each exposure category, or summary measures of exposure	-
		Cross-sectional study—Report numbers of outcome events or summary measures	9-10
Main results	16	(*a*) Give unadjusted estimates and, if applicable, confounder-adjusted estimates and their precision (eg, 95% confidence interval). Make clear which confounders were adjusted for and why they were included	9-10
		(*b*) Report category boundaries when continuous variables were categorized	9-10
		(*c*) If relevant, consider translating estimates of relative risk into absolute risk for a meaningful time period	9-10
Other analyses	17	Report other analyses done—eg analyses of subgroups and interactions, and sensitivity analyses	9-10
**Discussion**			
Key results	18	Summarise key results with reference to study objectives	10-11
Limitations	19	Discuss limitations of the study, taking into account sources of potential bias or imprecision. Discuss both direction and magnitude of any potential bias	13
Interpretation	20	Give a cautious overall interpretation of results considering objectives, limitations, multiplicity of analyses, results from similar studies, and other relevant evidence	14
Generalisability	21	Discuss the generalisability (external validity) of the study results	14
**Other information**			
Funding	22	Give the source of funding and the role of the funders for the present study and, if applicable, for the original study on which the present article is based	14

*Give information separately for cases and controls in case-control studies and, if applicable, for exposed and unexposed groups in cohort and cross-sectional studies.

Note: An Explanation and Elaboration article discusses each checklist item and gives methodological background and published examples of transparent reporting. The STROBE checklist is best used in conjunction with this article (freely available on the Web sites of PLoS Medicine at http://www.plosmedicine.org/, Annals of Internal Medicine at http://www.annals.org/, and Epidemiology at http://www.epidem.com/). Information on the STROBE Initiative is available at www.strobe-statement.org.

For peer review only
